# *Ex vivo* activation of CD4^+^ T-cells from donors on suppressive ART can lead to sustained production of infectious HIV-1 from a subset of infected cells

**DOI:** 10.1371/journal.ppat.1006230

**Published:** 2017-02-22

**Authors:** John K. Bui, Elias K. Halvas, Elizabeth Fyne, Michele D. Sobolewski, Dianna Koontz, Wei Shao, Brian Luke, Feiyu F. Hong, Mary F. Kearney, John W. Mellors

**Affiliations:** 1 Division of Infectious Diseases, Department of Medicine, University of Pittsburgh School of Medicine, Pittsburgh, Pennsylvania, United States of America; 2 Howard Hughes Medical Research Fellows Program, Howard Hughes Medical Institute, Bethesda, Maryland, United States of America; 3 Advanced Biomedical Computing Center, Frederick National Laboratory for Cancer Research operated by Leidos Biomedical Research, Inc., Frederick, Maryland, United States of America; 4 HIV Dynamics and Replication Program, National Cancer Institute, Frederick, Maryland, United States of America; John Hopkins University, UNITED STATES

## Abstract

The fate of HIV-infected cells after reversal of proviral latency is not well characterized. Simonetti, *et al*. recently showed that CD4^+^ T-cells containing intact proviruses can clonally expand *in vivo* and produce low-level infectious viremia. We hypothesized that reversal of HIV latency by activation of CD4^+^ T-cells can lead to the expansion of a subset of virus-producing cells rather than their elimination. We established an *ex vivo* cell culture system involving stimulation of CD4^+^ T-cells from donors on suppressive antiretroviral therapy (ART) with PMA/ionomycin (day 1–7), followed by rest (day 7–21), and then repeat stimulation (day 21–28), always in the presence of high concentrations of raltegravir and efavirenz to effectively block new cycles of viral replication. HIV DNA and virion RNA in the supernatant were quantified by qPCR. Single genome sequencing (SGS) of p6-PR-RT was performed to genetically characterize proviruses and virion-associated genomic RNA. The replication-competence of the virions produced was determined by the viral outgrowth assay (VOA) and SGS of co-culture supernatants from multiple time points. Experiments were performed with purified CD4^+^ T-cells from five consecutively recruited donors who had been on suppressive ART for > 2 years. In all experiments, HIV RNA levels in supernatant increased following initial stimulation, decreased or remained stable during the rest period, and increased again with repeat stimulation. HIV DNA levels did not show a consistent pattern of change. SGS of proviruses revealed diverse outcomes of infected cell populations, ranging from their apparent elimination to persistence and expansion. Importantly, a subset of infected cells expanded and produced infectious virus continuously after stimulation. These findings underscore the complexity of eliminating reservoirs of HIV-infected cells and highlight the need for new strategies to kill HIV-infected cells before they can proliferate.

## Author summary

Stable latent reservoirs of HIV persist despite suppressive antiretroviral therapy (ART) and cause rebound viremia following interruption of ART. New HIV cure strategies aim to deplete the latent reservoir by reversing HIV latency and promoting the death of cells containing inducible proviruses. Multiple latency reversing agents have been discovered, with the most effective compounds also causing T-cell activation. We investigated the *ex vivo* effects of cell activation on HIV-infected cells from individuals on stable suppressive ART. Latency reversal through robust cell activation led to diverse outcomes of infected cells. Although many infected cells appeared to be eliminated following T-cell activation, a subset of HIV-infected cells persisted and could expand, including those that can produce infectious virus. These findings highlight the need for new therapies that kill HIV-infected cells before they can proliferate.

## Introduction

The major barrier to curing HIV infection in individuals receiving suppressive antiretroviral therapy (ART) is a persistent viral reservoir consisting of infrequent cells that harbor latent, intact proviruses capable of being activated to produce infectious virus. It has been reported that the latent reservoir is primarily composed of long-lived resting CD4^+^ T-cells [1–4] that have a half-life of ~44 months [5]. The “shock-and-kill” strategy has been proposed as a means of depleting the HIV reservoir by reversing latency and promoting death of cells with reactivated proviruses by viral- or immune-mediated cytotoxicity [6]. Multiple latency reversing agents (LRAs) have been discovered but the most effective LRAs often induce T-cell activation [7–9].

Studies in untreated HIV infection have shown that the majority of productively infected cells undergo rapid cell death with a half-life of ~1 day [9–11], likely due to viral cytopathic effect (CPE) [12] and/or immune-mediated killing [13, 14]. However, Shan *et al*. used *in vitro* primary cell models of proviral latency to show that infected resting CD4^+^ T-cells are relatively resistant to viral cytopathic effect and can persist following latency reversal with vorinostat [10]. In addition, studies have shown that HIV-infected cells can persist and expand *in vivo* [15, 16]. In one patient, marked clonal expansion of cells with intact proviruses led to persistent viremia [17]. Given these findings, we hypothesized that a subset of inducible HIV-infected cells can persist and expand following T-cell activation.

To characterize the effects of T-cell activation on HIV-infected cells, we developed an *ex vivo* cell culture system involving stimulation of primary cells from chronically HIV-infected donors on suppressive ART with phorbol 12-myristate 13-acetate (PMA) and ionomycin. Single-genome sequencing (SGS) was used to study the dynamics of cells containing genetically distinct proviruses and the virions released into the supernatant. These experiments revealed that infected cell populations have several different outcomes following cellular activation and latency reversal. In contrast to previous findings [9–11], we observed that cells containing intact, inducible proviruses can persist and expand following cellular activation.

## Results

### Donor characteristics

Experiments were performed using unfractionated PBMC or total CD4^+^ T-cells purified from peripheral blood mononuclear cells (PBMC) obtained from five chronically HIV-1 infected donors on suppressive ART who met the eligibility criterion of having plasma HIV RNA ≤ 50 copies/mL for ≤ 2 years. The donors studied were the first five volunteers who met these eligibility criteria. Table 1 shows the characteristics of the five donors studied. The median age is 56 years (range 42–59 years); the median total number of years since detection of HIV seropositivity is 25 years (range 20–27 years); and, the median number of years of plasma viral HIV RNA suppression to ≤ 50 copies/mL on ART is 15 years (range 2–18 years).

**Table 1:** Characteristics of study subjects.

Donor	Age	Race	Gender	Years infected	Years suppressed	Pre-ART VL	Nadir CD4	Current CD4	Current Drug regimen
**1**	56	African American	Female	25	14	366,200	410	1505	EFV / FTC / TDF
**2**	42	African American	Male	20	2	—	210	524	FTC / RPV / TDF
**3**	59	African American	Male	22	18	117,068	314	1023	3TC / ABC / EFV
**4**	52	Caucasian	Male	27	18	99,985	153	585	3TC / ABC / RAL
**5**	57	African American	Female	25	15	13,048	13	930	EFV / FTC / TDF

3TC, lamivudine; ABC, abacavir; EFV, efavirenz; FTC, emtricitabine; RAL, raltegravir; TDF, tenofovir disoproxil fumarate.

**Fig 1:**
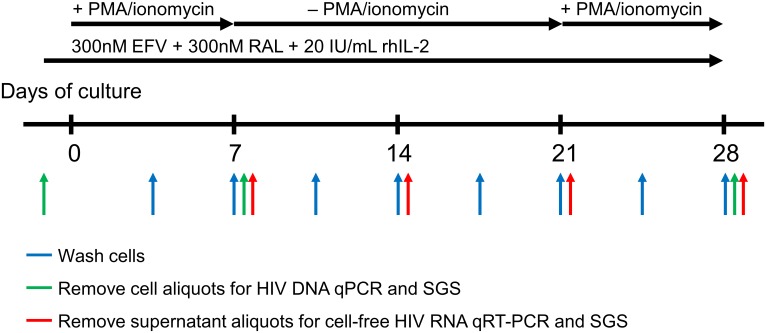
Experimental system schematic. PBMC or purified total CD4^+^ T-cells were cultured with 300 nM efavirenz (EFV), 300 nM raltegravir (RAL), and 20 IU/mL of recombinant human IL-2 (rhIL-2). Cells were sequentially stimulated with PMA (50 ng/mL) and ionomycin (500 ng/mL). The stimulation duration was seven days with an inter-stimulation period of fourteen days. Cells were washed twice weekly. Aliquots of cells from blood and from *ex vivo* cultures on days -1, 7, 21, and 28 were saved for HIV DNA qPCR and/or single-genome sequencing (SGS). Aliquots of supernatant were removed once weekly for virion-associated HIV RNA qRT-PCR and SGS.

Total CD4^+^ T-cells or PBMC were stimulated with two sequential 7-day exposures to PMA (50 ng/mL) and ionomycin (500 ng/mL) with a 14-day intervening period of non-exposure (Fig 1). Cells were incubated continuously with 300nM efavirenz and 300nM raltegravir to block viral replication (SGS analysis of virion-associated HIV RNA revealed no evidence of replication, as described below). Aliquots of cells and supernatant were taken at multiple time points for downstream analysis (Fig 1). Experiments were performed with total CD4^+^ T-cells for donors 1–5, with a repeat experiment also performed for donor 1. Experiments were performed with PBMC for donors 1 and 5.

### PMA and Ionomycin treatment achieves robust cellular activation

**Fig 2:**
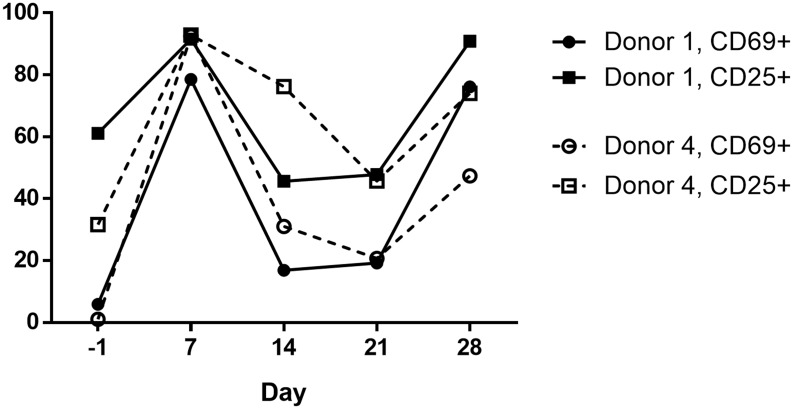
Activation marker expression during cell culture. The frequency of CD69^+^ and CD25^+^ cells is measured relative to the parent population of live, CD3^+^CD4^+^ lymphocytes.

The effects of PMA (50 ng/mL) and ionomycin (500 ng/mL) on expression of the activation markers CD69 and CD25 was measured on cells from weekly time points (Fig 2). The first week of PMA and ionomycin exposure induced activation in > 95% of cells. During the 14-day rest period, the frequency of activated T-cells returned to baseline levels. The second round of stimulation induced potent activation again, although in fewer cells for donor 4.

**Fig 3:**
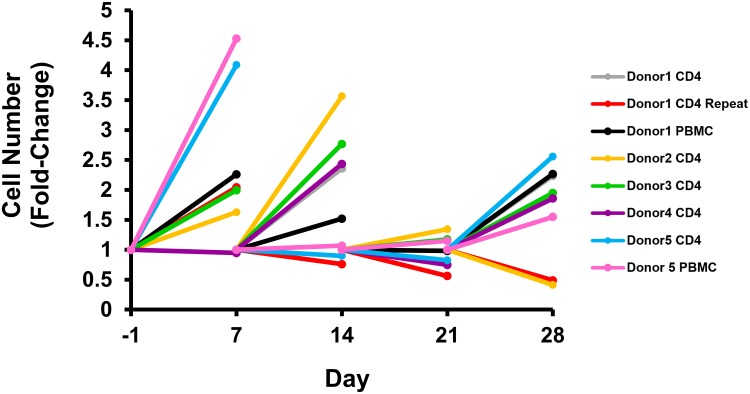
Cell number changes during cell culture. Cell numbers at the conclusion of each week are normalized relative to the total number of cells that were seeded in the flask at the beginning of each week.

Cell numbers were measured at the beginning and conclusion of each week and were normalized relative to the number of cells at the beginning of each week (Fig 3). During the first stimulation week, there was a median 2.0-fold increase in total CD4^+^ T-cells and a median 3.4-fold increase in PBMC. During the second stimulation week, there was a median 1.9-fold increase in total CD4^+^ T-cells and 1.9-fold increase in PBMC, respectively. The proliferation of cultured cells during the stimulation weeks confirms that PMA and ionomycin induced robust cell activation. A reduction in cell number was observed in two of six experiments with total CD4^+^ T-cells during the second stimulation, which is not unexpected from activation-induced cell death [18]. The two experiments performed using total CD4^+^ T-cells from donor 1 showed similar proliferation following the first stimulation, but showed greater variation at later time points. This likely reflects variability that can be seen with primary cell cultures over extended durations of culture.

### Changes in HIV DNA

**Fig 4:**
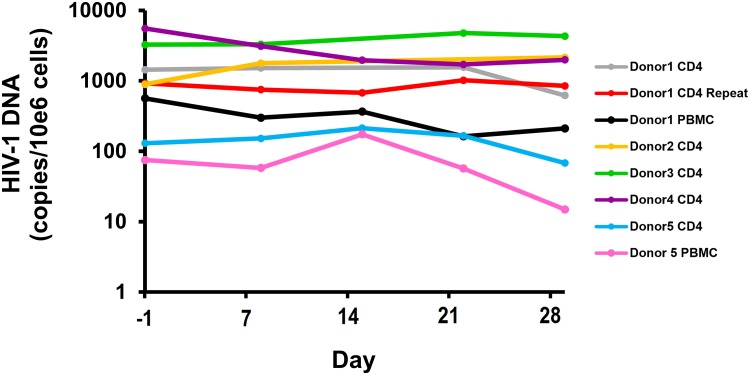
Levels of HIV DNA during cell culture. The frequency of HIV-infected cells was quantified using qPCR.

HIV DNA was quantified in cells by using qPCR targeting *pol* and normalizing this value to cell number as measured by qPCR targeting *CCR5* (Fig 4) [19]. Overall, no significant change in HIV DNA occurred in total CD4^+^ T-cells between baseline and post-stimulation time points at days 7 and 28 (Wilcoxon Signed Rank test, P > 0.05). When comparing baseline HIV DNA to day 7 HIV DNA in total CD4^+^ T-cells, HIV DNA decreased in two of six experiments (median 1.5-fold) and increased in four of six experiments (median 1.1-fold). When comparing baseline HIV DNA to day 28 HIV DNA in total CD4^+^ T-cells, HIV DNA decreased in four of six experiments (median 2.1-fold decrease) and increased in two of six experiments (median 1.9-fold increase). For the two experiments performed with PBMC, HIV DNA decreased by a median 1.5-fold from baseline to day 7, and by a median 3.5-fold from baseline to day 28. By using cell counts and HIV DNA measurements, the numbers of total cells and infected cells could be calculated and compared. Following the first stimulation, there was a median 2.0-fold increase in total CD4^+^ T-cells, 2.1-fold increase in infected CD4^+^ T-cells, 3.4-fold increase in total PBMC, and 2.4-fold increase in infected PBMC. Following the second stimulation, there was a median 1.9-fold increase in total CD4^+^ T-cells, 1.0-fold change in infected CD4^+^ T-cells, a 1.9-fold increase in total PBMC, and 1.7-fold change in infected PBMC. There was no significant difference between relative changes in cells numbers between total and infected cells for both CD4^+^ T-cells and PBMC (P > 0.5 by Wilcoxon matched-pairs signed rank test).

### HIV virion production

**Fig 5:**
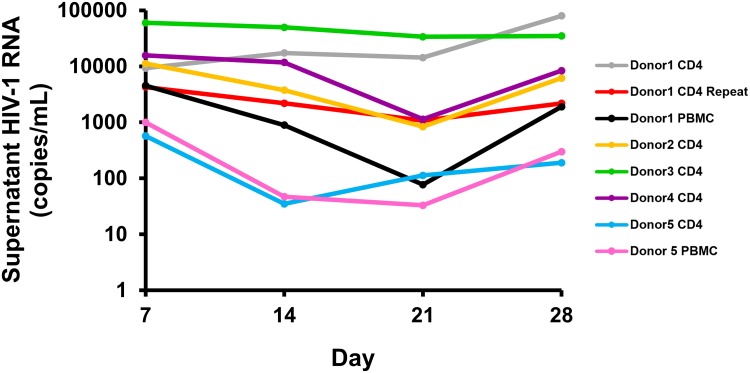
Levels of HIV virion production during cell culture. HIV virion production was measured on cell-free supernatants using the Roche COBAS AmpliPrep/TaqMan HIV-1 test. The cumulative levels of virus produced spontaneously after 24 hours in separate experiments were < 20 copies/mL.

HIV virion production was quantified by measuring HIV nucleic acid in cell culture supernatants using the Roche TaqMan 2.0 (Fig 5). Control amplifications with exclusion of reverse transcriptase [20] confirmed that quantified nucleic acid contained >95% HIV RNA. All donors had plasma HIV RNA < 20 copies/mL before entry. In preliminary experiments, primary total CD4^+^ T-cells that were cultured *ex vivo* for 24 hours without stimulation produced virions at quantities lower than the limit of detection (< 20 copies/mL). All experiments with total CD4^+^ T-cells showed virion production following the first stimulation week (median 10,261 HIV copies/mL), decreased or stable virion production was observed over the two rest weeks (median 4.4-fold decrease), and increased virion production following the second stimulation week (median 3.8-fold increase). The two experiments performed using total CD4^+^ T-cells from donor 1 showed similar virion production following the first stimulation, but exhibited greater variation at later time points. This variation is likely due to the complex cellular dynamics that can occur in bulk cell cultures of primary cells. The PBMC experiments showed similar trends as compared with the total CD4^+^ T-cell experiments, with increased virion production following the first stimulation (median 2,766 HIV copies/mL), decreased virion production over the two rest weeks (median 40-fold decrease), and increased virion production following the second stimulation week (median 17-fold increase).

Reduced virion production was observed following the second stimulation as compared to the first stimulation in all donors in both total CD4^+^ T-cells (median 1.8-fold reduction) and PBMC (median 2.8-fold reduction), except for one of the two experiments with donor 1 using total CD4^+^ T-cells (8.6-fold increase with the second stimulation).

### Dynamics of HIV-infected cell populations

#### Interpretation of Single-Genome Sequences (SGS)

To characterize cellular dynamics and virion production from HIV-infected cells, SGS of p6 of *gag*, *pro* and the first 900 nucleotides of *pol* (p6-PR-RT) [21, 22] was performed on HIV DNA in cultured cells and virion RNA in culture supernatants. When performing SGS on virion RNA, control reactions performed in the absence of reverse transcriptase were either negative or rarely positive with HIV DNA contamination estimated to be < 0.5% of nucleic acid in the extracted sample. No cross-contamination of proviral or virion sequences was detected across the different donors by neighbor-joining distance tree analysis (S1 Fig).

We used neighbor-joining distance analyses of single-genome sequences to investigate the expression of specific proviruses and the population dynamics of cells containing specific proviruses. An example of the neighbor-joining distance trees is shown in S2 Fig. Identical p6-PR-RT HIV DNA sequences at baseline suggests *in vivo* proliferation of HIV-infected cells. An increased frequency of a specific proviral sequence over time suggests *ex vivo* proliferation of cells containing a specific provirus. Conversely, a decreased frequency of a proviral sequence over time suggests *ex vivo* elimination of cells containing a specific provirus. HIV sequences in virions released into the culture supernatants reveal whether a specific provirus is inducible and when it was induced.

#### Interpretation of identical sequences

Although our targeted p6-PR-RT amplicon makes up only ~15% of the HIV proviral genome, the five donors had diverse proviral sequences with a median 1.5% average pairwise distance (APD) of all proviral sequences (S2 Table) that improve the ability to distinguish between different viral sequences [21–23]. An algorithm was developed to calculate the probability of identifying two identical sequences given the length of the sequence amplicon (S), the average pairwise distance between proviruses (APD), and the number of proviral sequences gathered (N) (see Materials and methods). These calculations showed that in the absence of cell proliferation there is less than a 1 in 700 chance of observing two identical proviral sequences when using the p6-PR-RT amplicon (S2 Table). The prolonged duration of untreated infection (Table 1) and the diverse proviral sequences indicate that these donors were not treated during acute infection. Therefore, identical HIV DNA sequences identified are likely due to cellular proliferation [23].

#### Summary of cellular and proviral outcomes

**Fig 6:**
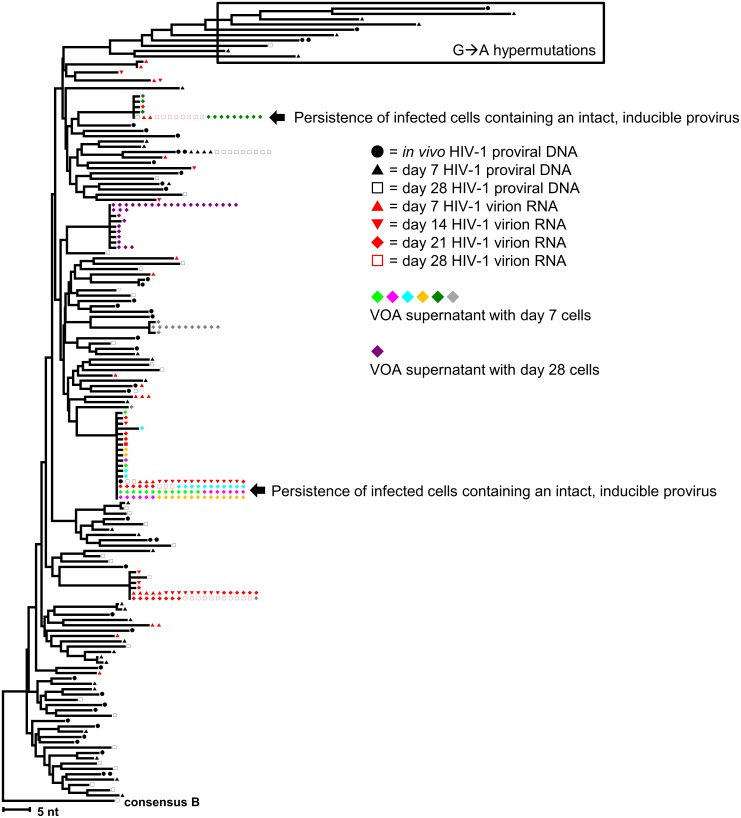
Neighbor-joining distance tree of virion-associated HIV RNA and proviral-associated HIV DNA sequences illustrates proviral population dynamics after CD4^+^ T-cell activation (Donor 1). The tree was constructed using the neighbor-joining p-distance method. All sequences were rooted to a consensus sequence of HIV subtype B. Hypermutant sequences are boxed. The Viral Outgrowth Assay (VOA) was performed using day 7 and day 28 cells from Donor 1 (Experiment 2) total CD4^+^ T-cells. The day 7 cells were seeded into 6 wells at 1x10^6^ cells/well and the day 28 cells were seeded into 6 wells at 3x10^5^ cells/well.

The findings from donor 1 are representative of the other four donors (Table 2). For donor 1, two phlebotomies were performed. The first phlebotomy was used to perform an experiment with total CD4^+^ T-cells (Fig 6) and the second phlebotomy was used to perform experiments with both total CD4^+^ T-cells (S3 Fig) and PBMC (S4 Fig). Before stimulation of total CD4^+^ T-cells from donor 1, a median of 4.4% of unique proviral sequences were observed to be identical. Out of all virion sequences that were detected over the duration of cell culture, 77.8% of unique virion sequences were detected only after the first stimulation and not the second stimulation. Approximately 5.6% of unique virion sequences were detected only after the second stimulation. Persistent expression of virions during both stimulations was observed for 16.7% of unique virion sequences.

**Table 2:** Proviral expression and dynamics in total CD4^+^ T-cells after sequential stimulation. Different proviral population outcomes are quantified for experiments with total CD4^+^ T-cells. Each outcome is calculated as either 1) the frequency of proviruses displaying a given outcome relative to the total number of unique proviral sequences observed over the entire duration of cell culture, or 2) the frequency of unique virion sequences displaying a given outcome relative to the total number of unique virion sequences observed over the entire duration of cell culture. IQR = interquartile range.

	Proviral Population Outcome	Donor 1	Donor 1 (repeat)	Donor 2	Donor 3	Donor 4	Donor 5	Median	IQR
**% of Unique Virion Sequences**	**Virions detected only after first stimulation**	**77.8%**	**88.5%**	**80.8%**	**60.0%**	**65.2%**	**83.3%**	**79.3%**	**68.4–82.7%**
	**Virions detected only after second stimulation**	**5.6%**	**3.8%**	**7.7%**	**20.0%**	**21.7%**	**16.7%**	**12.2%**	**6.1–19.2%**
	**Virions detected with both stimulations**	**16.7%**	**7.7%**	**11.5%**	**20.0%**	**13.0%**	**< 16.7%**	**12.3%**	**8.7–15.8%**
	***Ex vivo* persistence or expansion of intact, inducible proviruses**	**11.1%**	**7.7%**	**3.8%**	**5.0%**	**Not done**	**Not done**	**6.3%**	**4.7–8.5%**
**% of Unique Proviral Sequences**	***Ex vivo* expansion of non-induced proviruses**	**1.1%**	**3.8%**	**6.5%**	**4.9%**	**1.3%**	**3.5%**	**3.7%**	**1.9–4.7%**
	***Ex vivo* expansion of inducible proviruses**	**1.1%**	**< 1.3%**	**1.1%**	**1.2%**	**< 1.3%**	**1.8%**	**1.1%**	**0.3–1.2%**

*Ex vivo* clonal expansion was suggested by an increasing frequency of a proviral sequence after stimulation. We found proviral sequences that increased in frequency over time that matched virion sequences (1.1% of unique proviral sequences) and others that did not match virion sequences (1.1% of unique proviral sequences).

Similar infected cell population outcomes were observed in the second experiment with total CD4^+^ T-cells from donor 1 (S3 Fig, Table 2). An experiment was also performed using donor 1’s PBMC, which comprises more diverse cell types including CD8^+^ T-cells, NK cells, and B-cells. All the listed outcomes observed for total CD4^+^ T-cells were also observed for PBMC (S4 Fig, S1 Table).

Neighbor-joining distance trees from donors 1–5 are shown in Fig 6 and S3–S9 Figs. A summary of the observed proviral population outcomes is shown in Table 2 and S1 Table. Overall, we observed identical proviral sequences *in vivo* for all five donors. Most unique virion sequences were detected only following the first stimulation (median 79.3% in total CD4^+^ T-cells; median 53.9% in PBMC). No significant correlation was found between the fold-change in HIV DNA between day 0 and day 28 and the frequency of unique virion sequences that were only expressed following the first stimulation (Spearman ϱ = 0.59, P = 0.12). No changes in proviral diversity were detected over time (S3 Table), although this analysis is limited since most proviruses are defective [7, 24]. No correlation was observed between the number of virion particles produced and the average pairwise distances of virions produced at each time point (S4 Table) (Spearman P > 0.5), suggesting that the reduced virion production following the second stimulation was not solely due to the depletion of infected cells carrying inducible proviruses.

A minority of unique virion sequences were detected only following the second stimulation (median 12.2% in total CD4^+^ T-cells; median 35.5% in PBMC). Persistent virion production over the non-stimulation period was observed in all experiments (Fig 5). In experiments with total CD4^+^ T-cells, a median of 35.1% of unique virion sequences expressed during the rest weeks were also expressed during the first stimulation. In experiments with PBMC, this fraction was 25.0%. These persistently expresses viruses were oligoclonal (0–4 unique sequences). A median 35.1% of unique virion sequences detected during the rest period were also expressed during the first stimulation, indicating persistent expression of proviruses. The remaining fraction of unique virion sequences detected during the rest period was not detected during the first stimulation.

A minority of virion sequences were detected following both the first and second rounds of stimulation in four of five donors (median 12.3% in total CD4^+^ T-cells; median 10.5% in PBMC), suggesting the persistence of inducible proviral populations. Donor 5 did not show evidence of persistent virion expression in either total CD4^+^ T-cells or PBMC. However, this donor had a virion sequence that was detected following the first stimulation that matched a proviral sequence detected following the second stimulation, suggesting the persistence of a population of cells carrying inducible proviruses. For all donors, the persistently expressed virion sequences intermingled with the other virion sequences on the neighbor-joining trees.

Evidence for *ex vivo* clonal expansion of non-induced proviral populations was observed in five of five donors in total CD4^+^ T-cells (Fig 6, S3, S5–S8 Figs) and in one of two donors in PBMC (S4 Fig). Evidence suggesting *ex vivo* clonal expansion of inducible proviral populations was observed in three of five donors from total CD4^+^ T-cells (Fig 6, S5 and S6Figs) and in one of two donors from PBMC (S4 Fig).

#### Expansion of cells containing intact inducible proviruses

The replication-competence of virions produced from persistent and inducible proviruses was assessed by performing the VOA using aliquots of cultured total CD4^+^ T-cells from donors 1–3 and sequencing the virions from p24-positive wells. In all three donors, we found inducible proviral populations that persisted throughout the 28-day cell culture and had sequence matches to VOA sequences (arrows, Fig 6 and S3–S6 Figs). Furthermore, multiple lines of evidence suggest that clonal expansion of cells with intact proviruses occurred *in vivo* or *ex vivo* in three of three donors. In the first experiment with donor 1 total CD4^+^ T-cells, two distinct proviral sequences increased in frequency over time, matched supernatant virion sequences, and also matched VOA sequences from the second experiment, suggesting *ex vivo* clonal expansion of cells infected with inducible, intact proviruses. Also in donor 1, two distinct virion sequences that matched VOA sequences were found in both total CD4^+^ T-cell experiments as well as in the PBMC experiment, indicating *in vivo* clonal expansion of cells infected with inducible, intact proviruses. In donors 2 and 3, sequence matches were also found between VOA sequences and persistently detected virion sequences throughout the duration of cell culture, indicating the *in vivo* or *ex vivo* clonal expansion of cells containing inducible, intact proviruses and the persistence of these cells despite robust stimulation.

### No evidence of viral replication in treated cell culture system

Sequential stimulation experiments were performed in the presence of efavirenz (300nM) and raltegravir (300nM) to block viral replication. Proviral and virion sequences were analyzed for HIV drug resistance mutations using the HIV drug resistance databases from Stanford, the Rega Institute, and the Agence Nationale de Recherches sur le SIDA. Extensively hypermutated sequences, as determined by the online Hypermut algorithm (http://www.hiv.lanl.gov/content/sequence/HYPERMUT/hypermut.html), were excluded from the drug resistance analysis since these genomes were unlikely to be replication-competent [25]. Using the listed drug resistance databases, no proviral or virion sequences were predicted to be resistant to efavirenz, suggesting that HIV replication was successfully blocked by efavirenz. Drug resistance analysis for raltegravir was not performed since integrase (IN) was not included in the amplicon for SGS. It is unlikely that these donors contained raltegravir-resistant HIV strains because all donors, except for donor 4, were treatment-naïve to raltegravir. Donor 4 had been suppressed for three years on a combination ART regimen containing raltegravir, abacavir, and lamivudine at the time of phlebotomy, suggesting that this patient did not harbor raltegravir-resistant mutations [26].

Distance analysis was used to further assess whether ongoing viral replication was occurring during experiments. The average pairwise distances (APD) between induced virion sequences, with hypermutant sequences excluded, did not change over the duration of cell culture (P > 0.05 by Pearson and Spearman correlation) (S4 Table). Taken together, these results suggest that viral replication was not occurring *ex vivo*.

## Discussion

The most widely discussed approach to curing HIV infection is termed the “kick and kill” strategy [6], which aims to deplete the latent reservoir by reversing HIV latency and promoting the death of cells containing reactivated proviruses by viral cytopathic effect [9–11] or immune-mediated killing [13, 14]. Multiple latency reversing agents (LRAs) have been discovered, with the most effective agents possessing the quality of inducing T-cell activation [7–9]. To explore the effects of latency reversal by T-cell activation on the latent reservoir, we developed an *ex vivo* system involving sequential stimulation of primary total CD4^+^ T-cells or PBMC with PMA and ionomycin. We showed that this stimulation achieves robust cellular activation and latency reversal. We did not observe consistent changes in HIV DNA frequency across experiments. Virion production increased during the stimulation periods and decreased, but remained detectable, during the intervening period of non-exposure. Using SGS, we show that cells containing specific proviruses have diverse outcomes following stimulation, including their apparent elimination, persistence, or expansion. Importantly, a subset of inducible, replication-competent proviruses can persist and expand despite robust sequential stimulation.

We found identical p6-PR-RT *in vivo* proviral sequences in five of five donors, suggesting *in vivo* clonal expansion of infected cells. These findings are consistent with previous reports of clonal expansion being a common phenomenon in HIV-infected donors on suppressive ART [15, 16, 27]. *In vivo* clonal expansion of HIV proviruses can result from the proviral integration site, homeostatic mechanisms, or antigenic stimulation [15–17, 28].

Multiple outcomes for infected cells were observed. Many proviruses did not produce detectable virion with either stimulation, which is consistent with prior reports [7, 29, 30] and is expected given the high frequency of defective proviral genomes [29]. Most inducible proviral populations produced virions following the first but not the second stimulation, consistent with the death of cells containing activated proviruses [9–11]. Another possibility is that cells containing these proviruses became refractory to sequential stimulation due to T-cell exhaustion [31], as suggested by the reduced CD69 expression following the second round of stimulation as compared to the first. A minority of inducible proviral populations produced virions only following the second stimulation, similar to what has been observed previously [29]. This outcome may be attributed to stochastic latency reversal [29, 32]. We also observed persistence of inducible proviral populations following sequential stimulation in five of five donors.

Detectable virion production continued during the rest period in all experiments for all donors. Analyses of drug resistance mutations and genetic distances indicated that viral replication was not contributing to the persistent virion production. Virion sequences during this period were oligoclonal; a minority of these sequences arose from persistent expression of proviruses from the first stimulation whereas a majority of sequences were newly detected, likely a result of newly induced proviruses from persistent cell activation or from proviruses that were expressed during the stimulation period but were not detected by the assay at that time point.

Conflicting results have been published on whether HIV-infected cells with intact proviruses can undergo clonal expansion. Simonetti *et al*. [17] extensively characterized *in vivo* clonal expansion of an infectious HIV clone in a single patient. This clonal expansion likely resulted from persistent stimulation by tumor antigen, which was robust enough to cause increasing clone numbers over time despite the potential for viral cytopathic effect and immune-mediated killing. In contrast, Cohn *et al*. [27] found that 75 expanded T-cell clones from eight HIV-infected individuals had defective genomes and concluded that expanded clones cannot contain intact proviruses. However, the study design had limited sensitivity to detect clonal expansion of intact proviruses. Here, we show that cells containing intact and inducible proviruses can persist following robust stimulation *ex vivo*, despite the potential for viral cytopathic effect and immune-mediated killing as seen in our experiments with PBMC.

We found evidence suggesting the *ex vivo* persistence of cell populations carrying inducible proviruses in five of five donors and evidence suggesting *in vivo* or *ex vivo* clonal expansion of cells with intact proviruses in three of three donors. The ability for certain subsets of cells containing intact and inducible proviruses to persist and proliferate may be attributed to viral sequences, proviral integration sites, and cell types. The sequences of virions that were detected after both stimulations intermingled with other virion sequences on the neighbor-joining tree, suggesting that factors other than the viral sequences contributed to their persistence. Integration sites in cancer-associated genes have been shown to promote survival and clonal expansion of proviruses *in vivo* [15, 16]. In addition, HIV proviruses can be found in multiple T-cell subsets [28, 33, 34] that have heterogeneous survival and proliferative potential [35]. Identifying the proviral insertion sites and infected T-cell subsets that can give rise to expanded clones with intact proviruses is a high priority but is complicated both by the rarity of intact proviruses (~2% of proviruses are intact) [36] compared to the large majority of defective provirus and the overall low frequency of infected CD4+T-cells (0.1%-0.01%) [37].

Experiments performed in the presence of effector cells (CD8^+^ T-cells, B-cells, NK cells) did not affect the persistence of inducible, replication-competent proviruses and did not result in appreciable decreases in the concentration of HIV-infected cells. These findings may be consistent with the well-studied exhaustion of the innate and adaptive immune systems during chronic HIV infection [31, 38–40].

Potent latency reversal with T-cell activation using PMA and ionomycin did not significantly deplete the frequency of HIV-infected cells across all donors as measured by qPCR for HIV DNA. Changes in HIV DNA were inconsistent across donors (decreased in four experiments and increased in two experiments) suggesting that latency reversal with T-cell activation has variable effects on HIV-infected cells from different donors. The variable changes in HIV DNA may represent inter-donor variability in the survival of infected cells, possibly related to viral cytopathic effect, cell type, or proviral integration site. No significant change in cell proliferation was noted between total cell number and infected cells in both CD4^+^ T-cells and PBMC. Future studies will be needed to investigate the mechanisms behind the inter-donor variability in HIV DNA changes in response to latency reversal by T-cell activation.

Following repeat stimulation with PMA and ionomycin, we observed a trend toward reduced cellular proliferation and virion production after the second stimulation compared with the first, although these trends were not statistically significant. It is possible that the observed differences in proliferation could be due to differences in activation state prior to stimulation. However, pre-stimulation cellular activation levels were similar between the two stimulation periods and therefore likely did not play a major role in the observed response differences. One possible explanation for a reduction in virion production is cell death of a majority of infected-cells that were actively producing virus, as suggested by SGS. Another possibility is that the cells became exhausted following the first stimulation and consequently did not respond as strongly following the second stimulation. Following prolonged stimulation, T-cells have been observed to become exhausted and with progressive impairment of effector functions, including proliferative potential [31]. Whether the diminished virion production was from depletion of infected cells with inducible proviruses, reduced virion production from infected cells, or both would require single-cell analysis for elucidation [19, 41].

Some limitations of our study deserve mention. Our sample size was small (N = 5), which may limit the generalizability of our findings. However, it should be noted that the donors met broad eligibility criterion (plasma HIV RNA < 50 copies/mL for > 2 years) and were enrolled and studied consecutively, minimizing selection bias. In addition, five of five donors displayed evidence of persistence of inducible proviral populations after latency reversal from cell activation and three of three donors displayed evidence of persistence of inducible, intact proviral populations, making these observations relevant for chronically HIV-infected individuals on long-term suppressive ART. Nevertheless, our findings of persistence and expansion of specific proviruses following latency reversal with T-cell activation should be verified *in vivo*. Another limitation of our study is that IL-2 was added to cultures to promote cell viability. Preliminary experiments revealed that cell numbers precipitously declined during the inter-dose period if IL-2 was not provided, reflecting the current limitations of *ex vivo* cell culture with primary T-cells. Finally, we performed SGS on only a ~1.5kb portion of the HIV genome. Consequently, we did not prove that identical sequence matches were from proviruses that were identical throughout their genomes, but Poisson and binomial statistical analyses incorporating average pairwise differences of the proviruses in the regions sequenced indicated that this event was improbable (1 in 700 chance on average). Definitive evidence of clonal expansion requires integration site analysis to confirm identical integration sites between proviruses in different cells [15, 16, 27]; however, significant technological advances are need to show linkage of identical integration sites to identical proviral sequences for the rare infected cells with intact proviruses observed in the current experiments.

In summary, this study provides insight into the effects of latency reversal with T-cell activation on the latent HIV reservoir and exposes additional challenges when using these compounds toward achieving an HIV cure. We found that reversal of HIV latency by CD4^+^ T-cell activation results in diverse outcomes for proviral populations, ranging from their apparent elimination to expansion of proviruses capable of infectious virus production. Survival and expansion of a subset cells containing inducible HIV proviruses occurred after T-cell activation across multiple HIV-infected individuals in the absence or presence of autologous effector cells (e.g. CD8^+^ T-cells, NK cells, B-cells). Even if a net depletion of the latent reservoir occurs following cellular activation, some inducible, intact proviral populations may be able to persist. To effectively target these proviral populations, compounds that kill HIV-infected cells before cellular proliferation occurs will be needed.

## Materials and methods

### Isolation of total CD4^+^ T-cells from HIV-infected individuals on ART

Large volume phlebotomy (~180 mL) was performed on five, consecutive, HIV-infected donors who were on suppressive ART (<50 copies of HIV RNA/mL plasma) for ≥ 2 years (Table 1). All patients provided written informed consent and the blood donation protocol was approved by the University of Pittsburgh Institutional Review Board, PRO13070189 and PRO14120068. PBMC were isolated by Ficoll-Paque density gradient centrifugation. Next, total CD4^+^ T-cells were isolated by negative selection using the EasySep Human CD4+ T-cell Enrichment Kit (STEMCELL).

### Ex vivo sequential stimulation culture

Isolated total CD4^+^ T-cells or PBMC were resuspended in RPMI medium 1640 without phenol red containing 10% (vol/vol) fetal bovine serum, 0.6% penicillin/streptomycin, 300nM efavirenz, and 300nM raltegravir. The cells were then cultured in T75 cm^2^ flasks. Following one day of rest, the cells were stimulated for seven days with 50ng/mL PMA and 500ng/mL ionomycin. Following the seven days of stimulation, the cells were washed three times and then transferred to a new flask in fresh media to be cultured in the absence of PMA and ionomycin for seven days. After an additional seven days, the cells were washed three times, transferred to a new flask in fresh media, and cultured again in the absence of PMA and ionomycin for seven days. After the seven days, the cells were washed three times, transferred to a new flask in fresh media, and cultured in the presence of PMA and ionomycin for seven days.

On each day of transferring cells to a new flask, cell numbers were counted using a hemocytometer, aliquots of cells were removed and saved in liquid nitrogen, and aliquots of cells and supernatant were removed and stored at -80°C for downstream analysis. Three days after each seeding of cells into a new flask, the cell media was changed.

### Modified Viral Outgrowth Assay (VOA)

A non-quantitative VOA was performed by co-culturing fresh aliquots of cells from the *ex vivo* sequential stimulation culture with allogeneic, irradiated feeder cells and CD8-depleted blasts as previously described [42]. The VOA was performed for donors 1–3. Supernatants from wells that produced detectable p24 by ELISA (Alliance HIV-1 P24 Antigen ELISA Kit, Perkin Elmer) were stored at -80°C for subsequent single-genome sequencing.

### Quantification of supernatant HIV RNA and cellular HIV DNA

HIV virion production was measured by qRT-PCR using the Roche COBAS AmpliPrep/TaqMan v2.0 (limit of detection = 20 HIV copies/mL). HIV DNA was quantified as previously described [19]. For baseline samples, HIV DNA was measured in PBMC and normalized to the number of total CD3^+^CD4^+^ lymphocytes as measured by flow cytometry.

### Flow cytometry analysis

Cells were analyzed for surface activation marker expression using the following anti-human monoclonal antibodies for staining: CD3-V450 (UCHT1), CD4-APC-H7 (RPA-T4), CD69-APC (FN50), CD25-PE (M-A251), and HLA-DR-PerCP-Cy5.5 (G46-6). Flow cytometry was performed using a BD LSRII flow cytometer equipped with BD FACSDiva v8.0.1 software. Analysis was performed on live cells (as determined by LIVE/DEAD Fixable Aqua Dead Cell Stain by ThermoFisher Scientific) that were single lymphocytes (by forward and side scatter) and CD3^+^CD4^+^. At least 2x10^5^ total events were acquired for each analysis.

### Single-Genome Sequencing (SGS)

SGS was performed on culture supernatants and cells. Extraction of nucleic acid from supernatant was performed as previously described [21, 22], except with initial centrifugation at 5,300xg for 10 min at 4°C to remove debris. Nucleic acid was extracted from cells as previously described [19]. cDNA was synthesized using the SuperScript III First-Strand Synthesis System. Each cDNA synthesis reaction was performed with 5μL of supernatant extract, 5μL of 10mM deoxynucleotide triphosphates, and 5μL of 2μM reverse primer targeting pol (5’-CTATTAAGTATTTTGATGGGTCATAA-3’). Following denaturation at 65°C for 10 min, each sample was quenched on ice followed by addition of 10μL of 10X RT buffer, 20μL of 25mM MgCl_2_, 1μL of DTT, 17.5μL of molecular-grade water, 1μL of RNase-Out, and 0.5μL of SuperScript III RT. The samples were then incubated at 25°C for 10 min, 45°C for 40 min, 85°C for 10 min, and then at 4°C.

The cDNA for SGS was plated using a limiting dilution scheme and amplified using nested PCR to determine a cDNA dilution that yields ~30% positive PCR reactions. At this dilution, ~80% of positive PCR reactions contain only a single copy of HIV cDNA in the reaction according to Poisson statistics [21, 22]. The nested PCR amplified a ~1.5kb amplicon spanning p6 of *gag*, *pro*, and the first ~900 nucleotides of *RT* as previously described [21, 22]. Positive nested PCR product was detected using GelRed (Biotium).

Following identification of a cDNA dilution that yielded ~30% positive PCR reactions, cDNA was plated at that dilution to gather more PCR product. Positive PCR product was treated with 20U of Exonuclease I (Affymetrix) and 4U of Shrimp Alkaline Phosphatase (Affymetrix). Positive PCR product was then prepared with the following four sequencing primers: 5’-TGTTGGAAATGTGGAAAGGAAGGAC-3’, 5’-ATGGCCCAAAAGTTAAACAATGGC-3’, 5’-TTCTTCTGTCAATGGCCATTGTTTAAC-3’, 5’-TTGCCCAATTCAATTTTCCCACTAA-3’. Sequences were aligned and analyzed for quality using Sequencher 5.2.

### Analysis of HIV sequences

Phylogenetic analysis was performed using the Neighbor-joining p-distance method in the MEGA 5.2 software. Hypermutant sequences were determined by the online algorithm: http://www.hiv.lanl.gov/content/sequence/HYPERMUT/hypermut.html. The APD between proviruses was calculated in MEGA 6. Hypermutant sequences, as determined by the online Hypermut algorithm, were excluded from analysis to avoid erroneous elevation of the APD. To calculate the expected number of identical HIV sequence pairs (L_e_), the total number of sequence pair comparisons (T) is multiplied by the probability of a sequence pair comparison being identical (P(0)): L_e_ = T * P(0). The T can be derived as follows: T = N * (N-1) / 2. The probability of identical sequences, P(0), is obtained using the Poisson distribution: P(0) = e^-λ^. Here, the λ is the average number of sequence differences: λ = APD * S. The probability of observing two identical sequences (or one sequence pair match) by chance was calculated using the binomial distribution to calculate the cumulative probability of observing an identical sequence over T trials given the probability P(0) of observing two identical sequences.

### Data availability

Sequences were submitted to the GenBank database (accession numbers: KX829224-829753, KX830756-830801).

## Supporting information

S1 Fig Neighbor-joining tree of all donor HIV sequences shows no evidence of inter-donor sequence contamination. No intermingling of patient sequences was observed.
(TIF)

S2 Fig Neighbor-joining distance tree of HIV virion RNA and proviral DNA sequences from a hypothetical sequential stimulation experiment. (1) Identical p6-PR-RT HIV proviral DNA sequences at day 0 suggests *in vivo* proliferation of HIV-infected cells. (2) An increased frequency of a proviral sequence over time suggests *ex vivo* proliferation of a specific proviral population. (3) A decreased frequency of a proviral sequence observed over time suggests *ex vivo* elimination of cells containing a specific provirus. (4) Proviral sequences without recovery of matching HIV virion sequences suggests that these sequences were not inducible. HIV sequences in virions released into the culture supernatants reveal whether a specific provirus was inducible and whether it was induced following the first stimulation (5), the second stimulation (6), or with both stimulations (7).
(TIF)

S3 Fig Neighbor-joining distance tree for Donor 1 (Experiment 2): Total CD4^+^ T-cells. Sequences were rooted to a consensus sequence of HIV subtype B. The tree was constructed using the neighbor-joining p-distance method. Hypermutant sequences are in boxes. The Viral Outgrowth Assay was performed using day 7 and day 28 cells.
(TIF)

S4 Fig Neighbor-joining distance tree for Donor 1 (Experiment 2): PBMC. Sequences were rooted to a consensus sequence of HIV subtype B. The tree was constructed using the neighbor-joining p-distance method. Hypermutant sequences are in boxes. The Viral Outgrowth Assay (VOA) was performed using day 7 and day 28 cells from Donor 1 (Experiment 2) total CD4^+^ T-cells. The day 7 cells were seeded into 6 wells at 1x10^6^ cells/well and the day 28 cells were seeded into 6 wells at 3x10^5^ cells/well.
(TIF)

S5 Fig Neighbor-joining distance tree for Donor 2: Total CD4^+^ T-cells. Sequences were rooted to a consensus sequence of HIV subtype B. The tree was constructed using the neighbor-joining p-distance method. Hypermutant sequences are in boxes. The Viral Outgrowth Assay was performed using day 14 cells. The day 14 cells were seeded into 5 wells at 1x10^5^ cells/well.
(TIF)

S6 Fig Neighbor-joining distance tree for Donor 3: Total CD4^+^ T-cells. Sequences were rooted to a consensus sequence of HIV subtype B. The tree was constructed using the neighbor-joining p-distance method. Hypermutant sequences are in boxes. The Viral Outgrowth Assay was performed using cells from day 7, day 14, day 21, and day 28. The day 7 cells were seeded into 4 wells at 1.25x10^6^ cells/well; day 14 cells were seeded into 4 wells at 1.25x10^6^ cells/well; day 21 cells were seeded into 4 wells at 1.25x10^6^ cells/well; and day 28 cells were seeded into 4 wells at 1.25x10^6^ cells/well.
(TIF)

S7 Fig Neighbor-joining distance tree for Donor 4: Total CD4^+^ T-cells. Sequences were rooted to a consensus sequence of HIV subtype B. The tree was constructed using the neighbor-joining p-distance method. Hypermutant sequences are in boxes.
(TIF)

S8 Fig Neighbor-joining distance tree for Donor 5: Total CD4^+^ T-cells. Sequences were rooted to a consensus sequence of HIV subtype B. The tree was constructed using the neighbor-joining p-distance method. Hypermutant sequences are in boxes.
(TIF)

S9 Fig Neighbor-joining distance tree for Donor 5: PBMC. Sequences were rooted to a consensus sequence of HIV subtype B. The tree was constructed using the neighbor-joining p-distance method. Hypermutant sequences are in boxes.
(TIF)

S1 Table Proviral expression and dynamics in PBMC after sequential stimulation. Different proviral population outcomes are quantified for experiments with PBMC. Each outcome is calculated as either 1) the frequency of proviruses displaying a given outcome relative to the total number of unique proviral sequences observed over the entire duration of cell culture, or 2) the frequency of unique virion sequences displaying a given outcome relative to the total number of unique virion sequences observed over the entire duration of cell culture.
(DOCX)

S2 Table Probability estimate of detecting two identical proviral sequences. The probability of detecting two identical p6-PR-RT sequences assuming no clonal expansion had occurred was calculated for each experiment using the binomial distribution based on the average pairwise distance (APD) of all obtained proviral sequences for each experiment. Hypermutant sequences were excluded from analysis.
(DOCX)

S3 Table Average Pairwise Distances (APD) of proviral DNA sequences. Hypermutant sequences were excluded from analysis. N/A = not applicable because < 5 sequences recovered.
(DOCX)

S4 Table Average Pairwise Distances (APD) of supernatant viral RNA sequences. Hypermutant sequences were excluded from analysis. N/A = not applicable because < 5 sequences recovered.
(DOCX)

S5 Table Raw data for experiments with total CD4+ T-cells. (XLSX)

S6 Table Raw data for experiments with PBMC. (XLSX)

## References

[1] Finzi D, Hermankova M, Pierson T, Carruth LM, Buck C, Chaisson RE, et al Identification of a reservoir for HIV-1 in patients on highly active antiretroviral therapy. Science. 1997;278(5341):1295–300. 9360927 10.1126/science.278.5341.1295

[2] Wong JK, Hezareh M, Gunthard HF, Havlir DV, Ignacio CC, Spina CA, et al Recovery of replication-competent HIV despite prolonged suppression of plasma viremia. Science. 1997;278(5341):1291–5. 9360926 10.1126/science.278.5341.1291

[3] Finzi D, Blankson J, Siliciano JD, Margolick JB, Chadwick K, Pierson T, et al Latent infection of CD4+ T cells provides a mechanism for lifelong persistence of HIV-1, even in patients on effective combination therapy. Nature Medicine. 1999;5(5):512–7. 10.1038/8394 10229227

[4] Chun TW, Carruth L, Finzi D, Shen X, DiGiuseppe JA, Taylor H, et al Quantification of latent tissue reservoirs and total body viral load in HIV-1 infection. Nature. 1997;387(6629):183–8. 10.1038/387183a0 9144289

[5] Siliciano JD, Kajdas J, Finzi D, Quinn TC, Chadwick K, Margolick JB, et al Long-term follow-up studies confirm the stability of the latent reservoir for HIV-1 in resting CD4+ T cells. Nature Medicine. 2003;9(6):727–8. 10.1038/nm880 12754504

[6] Archin NM, Margolis DM. Emerging strategies to deplete the HIV reservoir. Current Opinion in Infectious Diseases. 2014;27(1):29–35. 10.1097/QCO.0000000000000026 24296585 PMC4031321

[7] Cillo AR, Sobolewski MD, Bosch RJ, Fyne E, Piatak M Jr., Coffin JM, et al Quantification of HIV-1 latency reversal in resting CD4+ T cells from patients on suppressive antiretroviral therapy. Proceedings of the National Academy of Sciences of the United States of America. 2014;111(19):7078–83. 10.1073/pnas.1402873111 24706775 PMC4024870

[8] Bullen CK, Laird GM, Durand CM, Siliciano JD, Siliciano RF. New ex vivo approaches distinguish effective and ineffective single agents for reversing HIV-1 latency in vivo. Nature Medicine. 2014;20(4):425–9. 10.1038/nm.3489 24658076 PMC3981911

[9] Spina CA, Anderson J, Archin NM, Bosque A, Chan J, Famiglietti M, et al An in-depth comparison of latent HIV-1 reactivation in multiple cell model systems and resting CD4+ T cells from aviremic patients. PLoS Pathogens. 2013;9(12):e1003834 10.1371/journal.ppat.1003834 24385908 PMC3873446

[10] Shan L, Deng K, Shroff NS, Durand CM, Rabi SA, Yang HC, et al Stimulation of HIV-1-specific cytolytic T lymphocytes facilitates elimination of latent viral reservoir after virus reactivation. Immunity. 2012;36(3):491–501. 10.1016/j.immuni.2012.01.014 22406268 PMC3501645

[11] Bolton DL, Hahn BI, Park EA, Lehnhoff LL, Hornung F, Lenardo MJ. Death of CD4(+) T-cell lines caused by human immunodeficiency virus type 1 does not depend on caspases or apoptosis. Journal of Virology. 2002;76(10):5094–107. 10.1128/JVI.76.10.5094-5107.2002 11967325 PMC136143

[12] Shedlock DJ, Hwang D, Choo AY, Chung CW, Muthumani K, Weiner DB. HIV-1 viral genes and mitochondrial apoptosis. Apoptosis: an International Journal on Programmed Cell Death. 2008;13(9):1088–99. 18622704 10.1007/s10495-008-0239-0

[13] Demers KR, Reuter MA, Betts MR. CD8(+) T-cell effector function and transcriptional regulation during HIV pathogenesis. Immunological Reviews. 2013;254(1):190–206. 10.1111/imr.12069 23772621 PMC3693771

[14] Deng K, Pertea M, Rongvaux A, Wang L, Durand CM, Ghiaur G, et al Broad CTL response is required to clear latent HIV-1 due to dominance of escape mutations. Nature. 2015;517(7534):381–5. 10.1038/nature14053 25561180 PMC4406054

[15] Maldarelli F, Wu X, Su L, Simonetti FR, Shao W, Hill S, et al HIV latency. Specific HIV integration sites are linked to clonal expansion and persistence of infected cells. Science. 2014;345(6193):179–83. 10.1126/science.1254194 24968937 PMC4262401

[16] Wagner TA, McLaughlin S, Garg K, Cheung CY, Larsen BB, Styrchak S, et al HIV latency. Proliferation of cells with HIV integrated into cancer genes contributes to persistent infection. Science. 2014;345(6196):570–3. 10.1126/science.1256304 25011556 PMC4230336

[17] Simonetti FR, Sobolewski MD, Fyne E, Shao W, Spindler J, Hattori J, et al Clonally expanded CD4+ T cells can produce infectious HIV-1 in vivo. Proceedings of the National Academy of Sciences of the United States of America. 2016. 10.1073/pnas.1522675113 PMC4763755 26858442

[18] Kottilil S, Bowmer MI, Trahey J, Howley C, Gamberg J, Grant MD. Fas/FasL-independent activation-induced cell death of T lymphocytes from HIV-infected individuals occurs without DNA fragmentation. Cellular Immunology. 2001;214(1):1–11. 10.1006/cimm.2001.1876 11902824

[19] Bui JK, Mellors JW, Cillo AR. HIV-1 virion production from single inducible proviruses following T-cell activation ex vivo. Journal of Virology. 2015. 10.1128/JVI.02520-15 PMC4719601 26559835

[20] Cillo AR, Vagratian D, Bedison MA, Anderson EM, Kearney MF, Fyne E, et al Improved single-copy assays for quantification of persistent HIV-1 viremia in patients on suppressive antiretroviral therapy. Journal of Clinical Microbiology. 2014;52(11):3944–51. 10.1128/JCM.02060-14 25187636 PMC4313209

[21] Palmer S, Kearney M, Maldarelli F, Halvas EK, Bixby CJ, Bazmi H, et al Multiple, linked human immunodeficiency virus type 1 drug resistance mutations in treatment-experienced patients are missed by standard genotype analysis. Journal of Clinical Microbiology. 2005;43(1):406–13. 10.1128/JCM.43.1.406-413.2005 15635002 PMC540111

[22] Kearney M, Palmer S, Maldarelli F, Shao W, Polis MA, Mican J, et al Frequent polymorphism at drug resistance sites in HIV-1 protease and reverse transcriptase. AIDS. 2008;22(4):497–501. 10.1097/QAD.0b013e3282f29478 18301062 PMC2921824

[23] Maldarelli F, Kearney M, Palmer S, Stephens R, Mican J, Polis MA, et al HIV populations are large and accumulate high genetic diversity in a nonlinear fashion. Journal of Virology. 2013;87(18):10313–23. 10.1128/JVI.01225-12 23678164 PMC3754011

[24] Bruner KM, Hosmane NN, Siliciano RF. Towards an HIV-1 cure: measuring the latent reservoir. Trends in Microbiology. 2015;23(4):192–203. 10.1016/j.tim.2015.01.013 25747663 PMC4386620

[25] Desimmie BA, Delviks-Frankenberrry KA, Burdick RC, Qi D, Izumi T, Pathak VK. Multiple APOBEC3 restriction factors for HIV-1 and one Vif to rule them all. Journal of Molecular Biology. 2014;426(6):1220–45. 10.1016/j.jmb.2013.10.033 24189052 PMC3943811

[26] Liedtke MD, Tomlin CR, Lockhart SM, Miller MM, Rathbun RC. Long-term efficacy and safety of raltegravir in the management of HIV infection. Infect Drug Resist. 2014;7:73–84. 10.2147/IDR.S40168 24672249 PMC3965364

[27] Cohn LB, Silva IT, Oliveira TY, Rosales RA, Parrish EH, Learn GH, et al HIV-1 integration landscape during latent and active infection. Cell. 2015;160(3):420–32. 10.1016/j.cell.2015.01.020 25635456 PMC4371550

[28] Chomont N, El-Far M, Ancuta P, Trautmann L, Procopio FA, Yassine-Diab B, et al HIV reservoir size and persistence are driven by T cell survival and homeostatic proliferation. Nature Medicine. 2009;15(8):893–900. 10.1038/nm.1972 19543283 PMC2859814

[29] Ho YC, Shan L, Hosmane NN, Wang J, Laskey SB, Rosenbloom DI, et al Replication-competent noninduced proviruses in the latent reservoir increase barrier to HIV-1 cure. Cell. 2013;155(3):540–51. 10.1016/j.cell.2013.09.020 24243014 PMC3896327

[30] Procopio FA, Fromentin R, Kulpa DA, Brehm JH, Bebin AG, Strain MC, et al A Novel Assay to Measure the Magnitude of the Inducible Viral Reservoir in HIV-infected Individuals. EBioMedicine. 2015;2(8):872–81. 10.1016/j.ebiom.2015.06.019 PMC4563128 26425694

[31] Wherry EJ, Kurachi M. Molecular and cellular insights into T cell exhaustion. Nature Reviews Immunology. 2015;15(8):486–99. 10.1038/nri3862 26205583 PMC4889009

[32] Dar RD, Hosmane NN, Arkin MR, Siliciano RF, Weinberger LS. Screening for noise in gene expression identifies drug synergies. Science. 2014;344(6190):1392–6. 10.1126/science.1250220 24903562 PMC4122234

[33] Brenchley JM, Hill BJ, Ambrozak DR, Price DA, Guenaga FJ, Casazza JP, et al T-cell subsets that harbor human immunodeficiency virus (HIV) in vivo: implications for HIV pathogenesis. Journal of Virology. 2004;78(3):1160–8. 10.1128/JVI.78.3.1160-1168.2004 14722271 PMC321406

[34] Buzon MJ, Sun H, Li C, Shaw A, Seiss K, Ouyang Z, et al HIV-1 persistence in CD4+ T cells with stem cell-like properties. Nature Medicine. 2014;20(2):139–42. 10.1038/nm.3445 24412925 PMC3959167

[35] Weng NP, Araki Y, Subedi K. The molecular basis of the memory T cell response: differential gene expression and its epigenetic regulation. Nature Reviews Immunology. 2012;12(4):306–15. 10.1038/nri3173 22421787 PMC4686144

[36] Bruner KM, Murray AJ, Pollack RA, Soliman MG, Laskey SB, Capoferri AA, et al Defective proviruses rapidly accumulate during acute HIV-1 infection. Nature Medicine. 2016. 10.1038/nm.4156 PMC5014606 27500724

[37] Hong F, Aga E, Cillo AR, Yates AL, Besson G, Fyne E, et al Novel Assays for Measurement of Total Cell-Associated HIV-1 DNA and RNA. Journal of Clinical Microbiology. 2016;54(4):902–11. 10.1128/JCM.02904-15 26763968 PMC4809955

[38] Ansari AW, Ahmad F, Meyer-Olson D, Kamarulzaman A, Jacobs R, Schmidt RE. Natural killer cell heterogeneity: cellular dysfunction and significance in HIV-1 immuno-pathogenesis. Cellular and Molecular Life Sciences: CMLS. 2015;72(16):3037–49. 10.1007/s00018-015-1911-5 25939268 PMC11113101

[39] Moir S, Fauci AS. B-cell exhaustion in HIV infection: the role of immune activation. Current Opinion in HIV and AIDS. 2014;9(5):472–7. 10.1097/COH.0000000000000092 25023621

[40] Bui JK, Mellors JW. Reversal of T-cell exhaustion as a strategy to improve immune control of HIV-1. AIDS. 2015;29(15):1911–5. 10.1097/QAD.0000000000000788 26355569

[41] Hataye JM, Casazza JP, Ambrozak D, Boritz E, Yamamoto T, Douek DC, et al. Sustained HIV Release by Single Persisting CD4+ T Cells During Latency Disruption. Conference on Retroviruses and Opportunistic Infections (CROI) 2016; February 22–25, 2016; Boston, Massachusetts2016.

[42] Siliciano JD, Siliciano RF. Enhanced culture assay for detection and quantitation of latently infected, resting CD4+ T-cells carrying replication-competent virus in HIV-1-infected individuals. Methods in Molecular Biology. 2005;304:3–15. 10.1385/1-59259-907-9:003 16061962

