# Supplementary figures and images for: Ex vivo activation of CD4+ T-cells from donors on suppressive ART can lead to sustained production of infectious HIV-1 from a subset of infected cells
Source: PLoS Pathog. 2017 Feb 22;13(2):e1006230. doi: 10.1371/journal.ppat.1006230 (PMC5338860; doi:10.1371/journal.ppat.1006230)

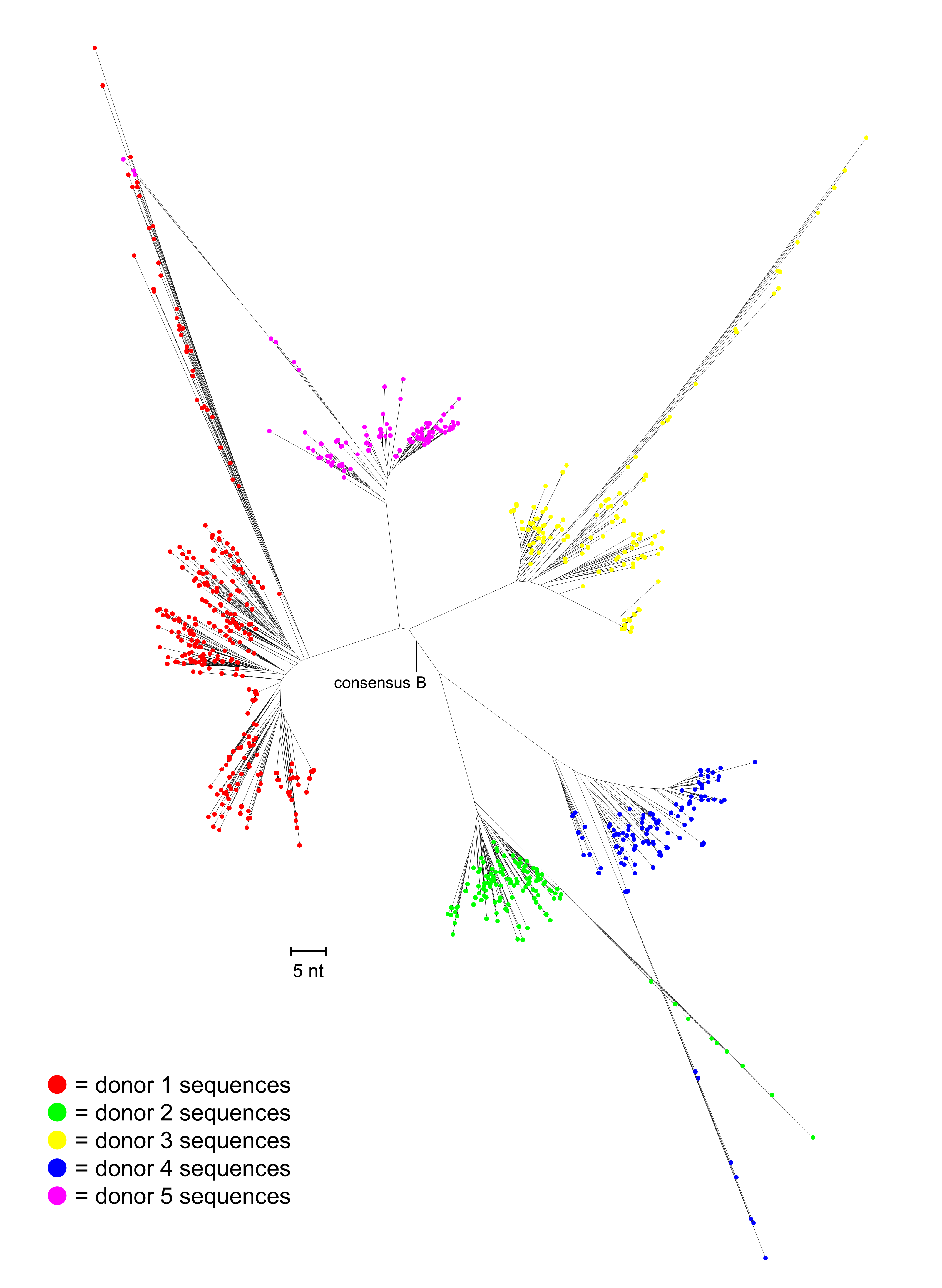

Supplement: S1 Fig — No intermingling of patient sequences was observed. (TIF) [file ppat.1006230.s001.TIF]

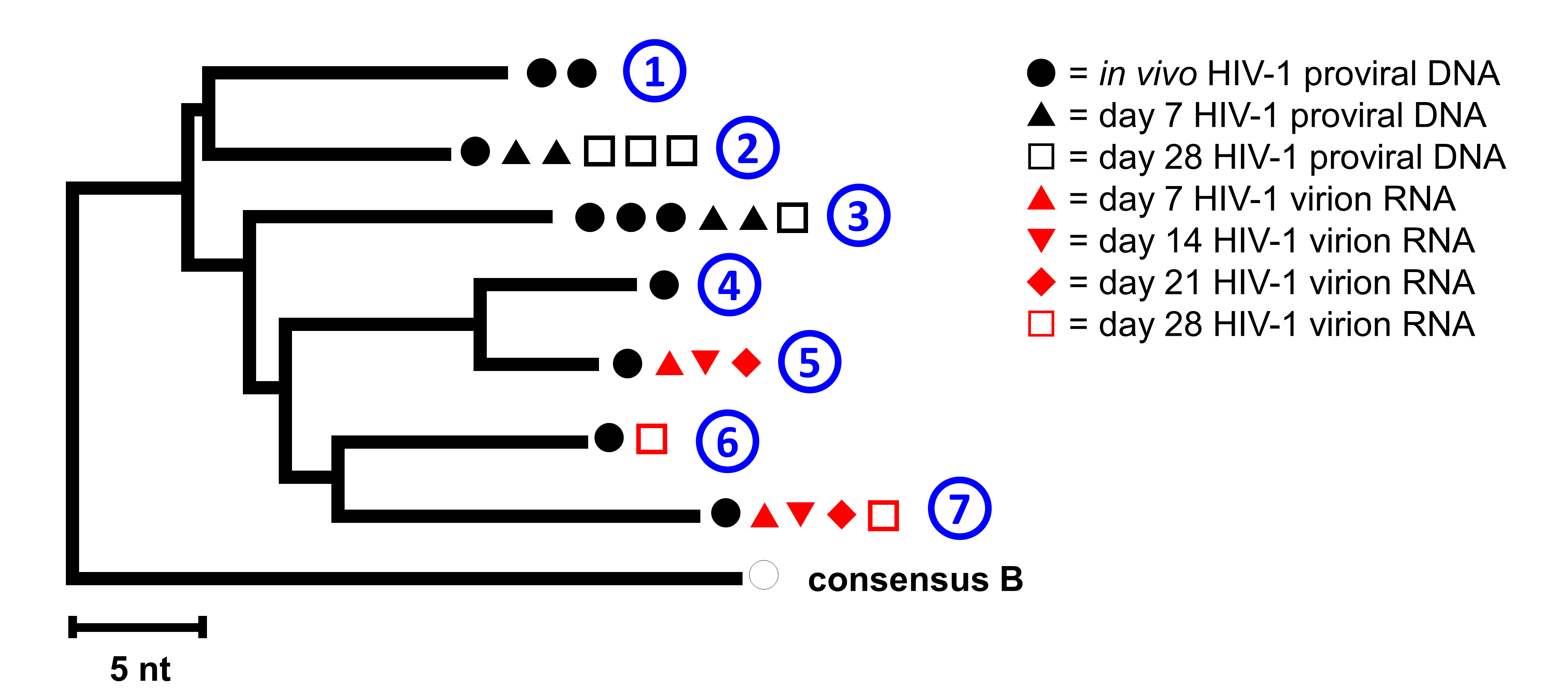

Supplement: S2 Fig — (1) Identical p6-PR-RT HIV proviral DNA sequences at day 0 suggests in vivo proliferation of HIV-infected cells. (2) An increased frequency of a proviral sequence over time suggests ex vivo proliferation of a specific proviral population. (3) A decreased frequency of a proviral sequence observed over time suggests ex vivo elimination of cells containing a specific provirus. (4) Proviral sequences without recovery of matching HIV virion sequences suggests that these sequences were not inducible. HIV sequences in virions released into the culture supernatants reveal whether a specific provirus was inducible and whether it was induced following the first stimulation (5), the second stimulation (6), or with both stimulations (7). (TIF) [file ppat.1006230.s002.TIF]

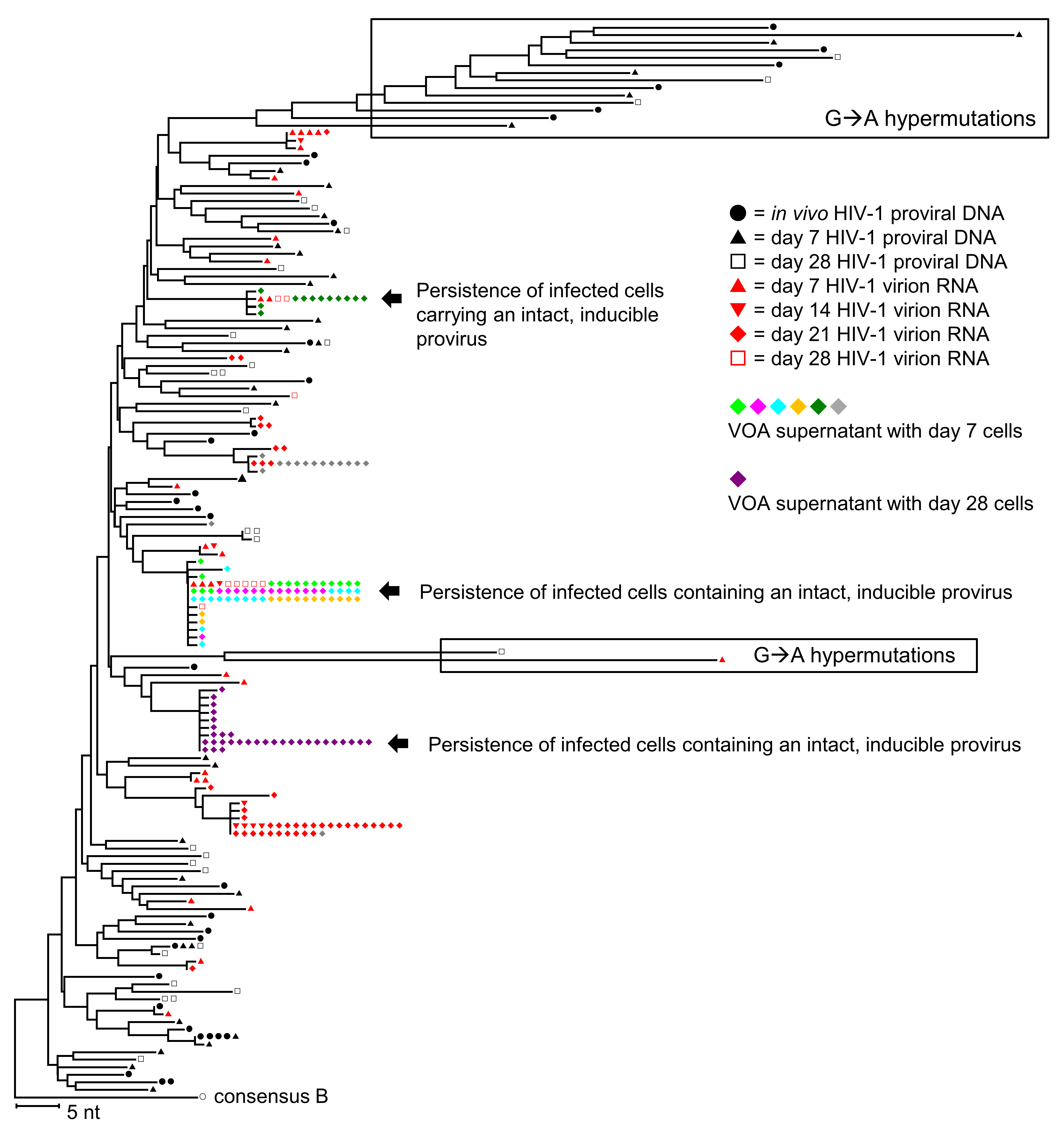

Supplement: S3 Fig — Sequences were rooted to a consensus sequence of HIV subtype B. The tree was constructed using the neighbor-joining p-distance method. Hypermutant sequences are in boxes. The Viral Outgrowth Assay was performed using day 7 and day 28 cells. (TIF) [file ppat.1006230.s003.TIF]

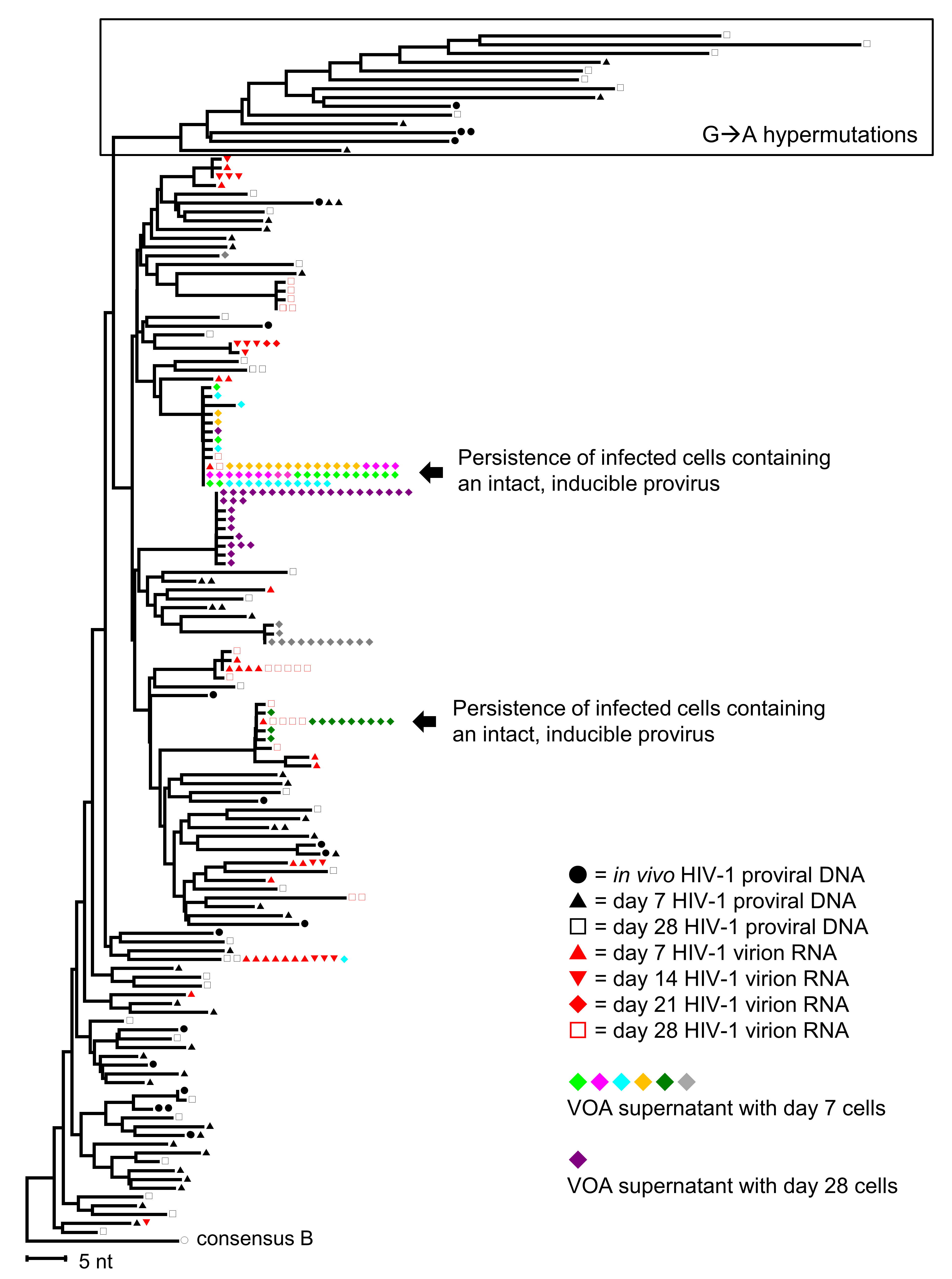

Supplement: S4 Fig — Sequences were rooted to a consensus sequence of HIV subtype B. The tree was constructed using the neighbor-joining p-distance method. Hypermutant sequences are in boxes. The Viral Outgrowth Assay (VOA) was performed using day 7 and day 28 cells from Donor 1 (Experiment 2) total CD4+ T-cells. The day 7 cells were seeded into 6 wells at 1x10^6^ cells/well and the day 28 cells were seeded into 6 wells at 3x10^5^ cells/well. (TIF) [file ppat.1006230.s004.TIF]

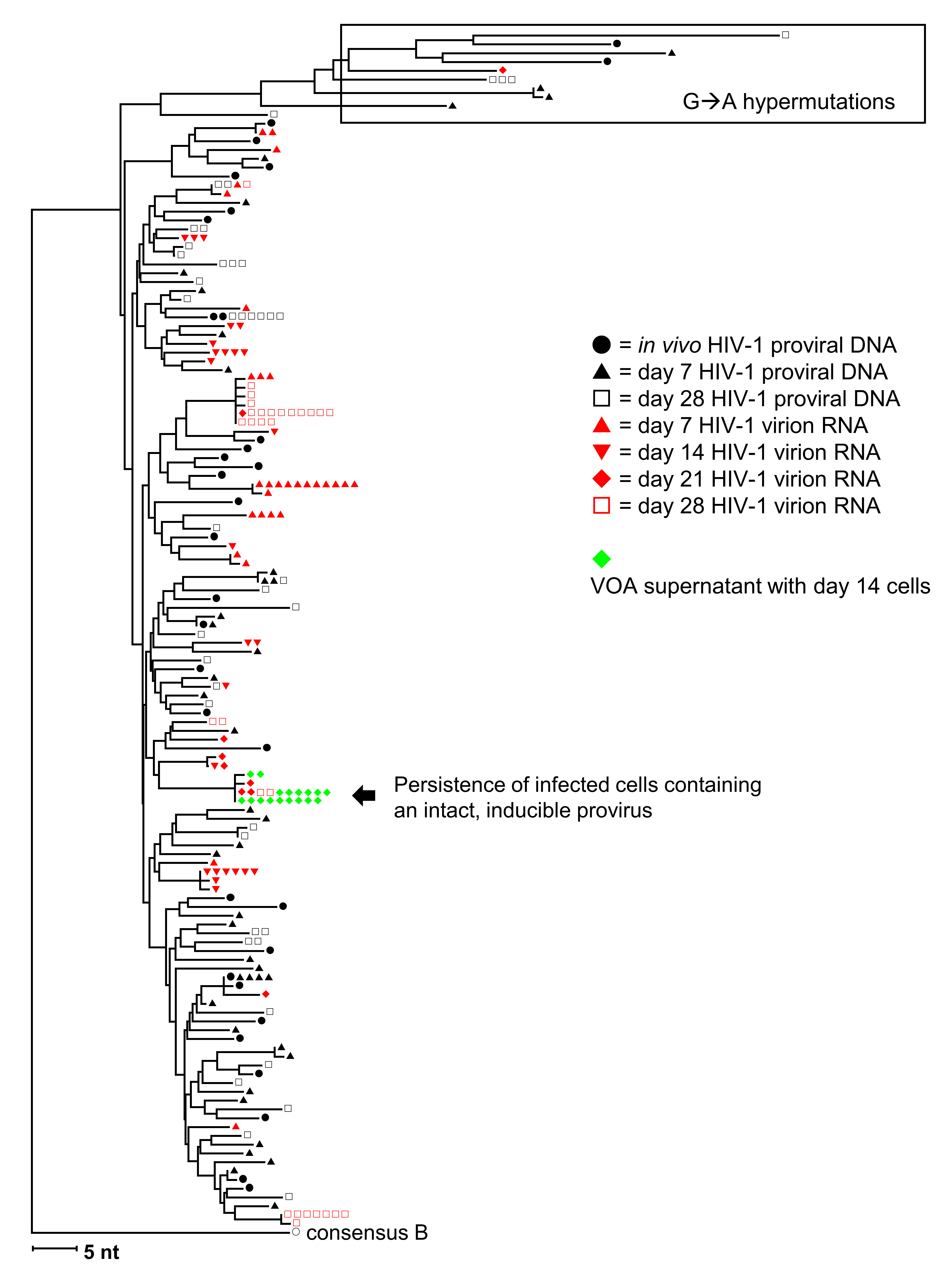

Supplement: S5 Fig — Sequences were rooted to a consensus sequence of HIV subtype B. The tree was constructed using the neighbor-joining p-distance method. Hypermutant sequences are in boxes. The Viral Outgrowth Assay was performed using day 14 cells. The day 14 cells were seeded into 5 wells at 1x10^5^ cells/well. (TIF) [file ppat.1006230.s005.TIF]

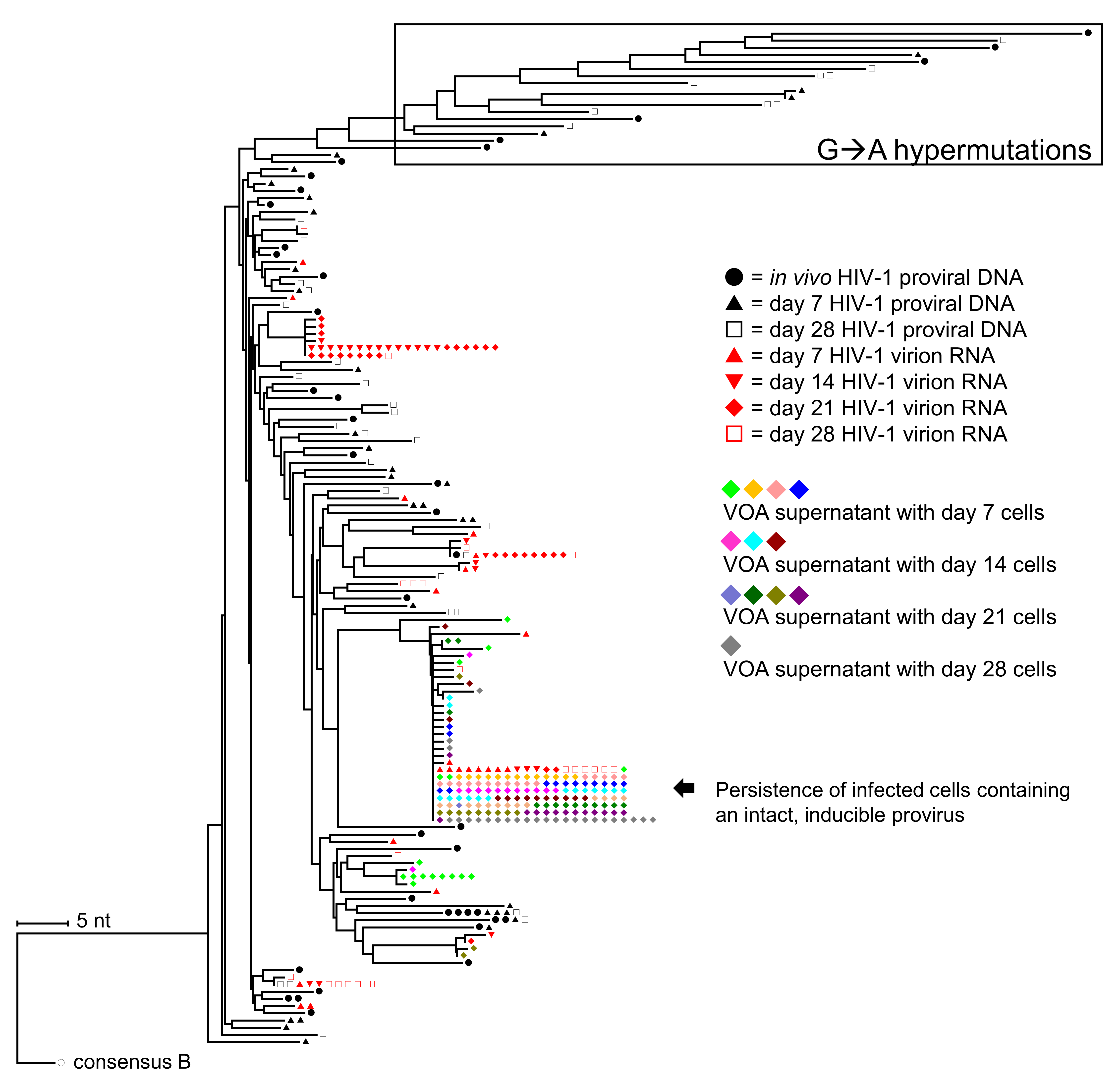

Supplement: S6 Fig — Sequences were rooted to a consensus sequence of HIV subtype B. The tree was constructed using the neighbor-joining p-distance method. Hypermutant sequences are in boxes. The Viral Outgrowth Assay was performed using cells from day 7, day 14, day 21, and day 28. The day 7 cells were seeded into 4 wells at 1.25x10^6^ cells/well; day 14 cells were seeded into 4 wells at 1.25x10^6^ cells/well; day 21 cells were seeded into 4 wells at 1.25x10^6^ cells/well; and day 28 cells were seeded into 4 wells at 1.25x10^6^ cells/well. (TIF) [file ppat.1006230.s006.TIF]

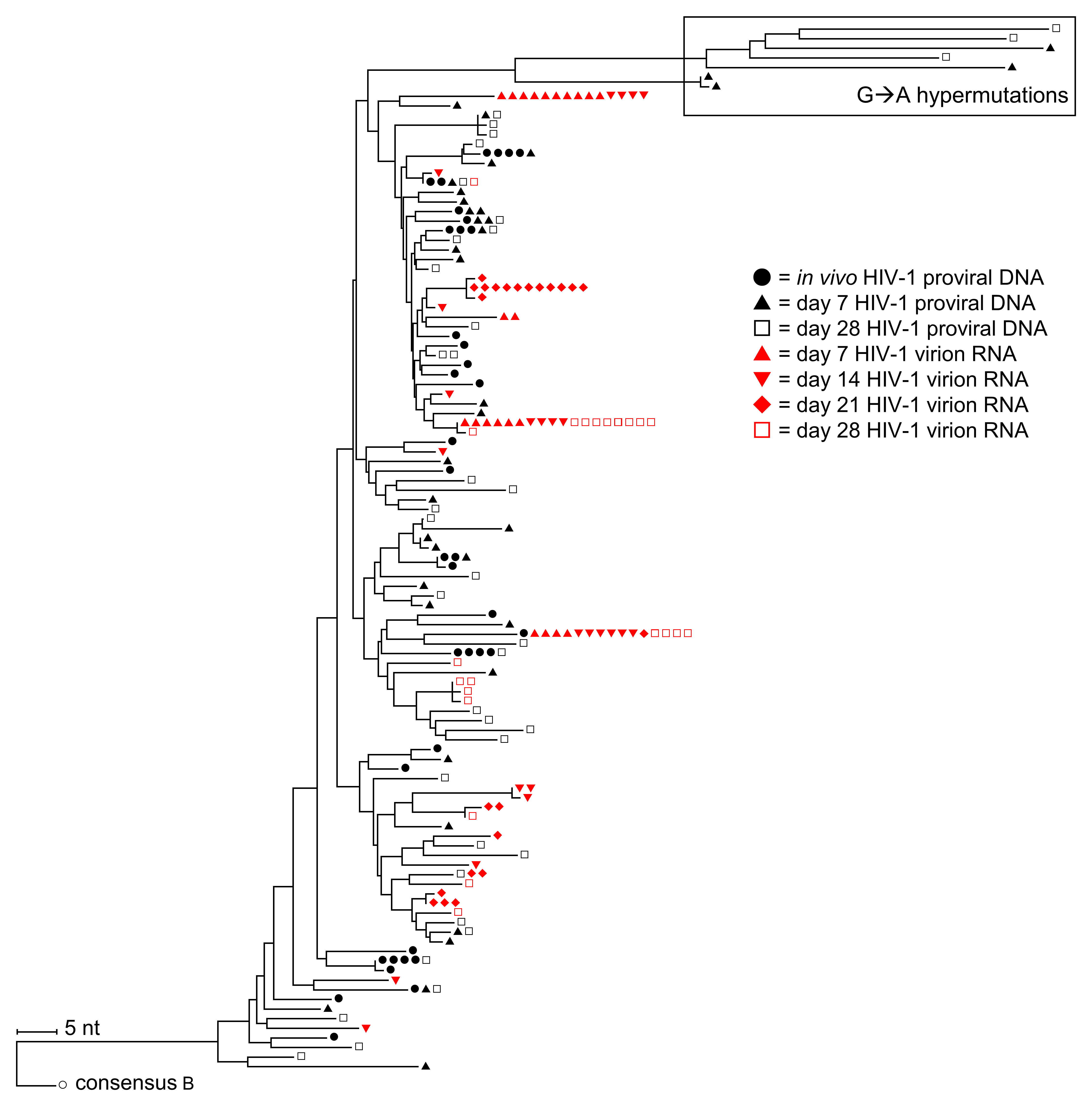

Supplement: S7 Fig — Sequences were rooted to a consensus sequence of HIV subtype B. The tree was constructed using the neighbor-joining p-distance method. Hypermutant sequences are in boxes. (TIF) [file ppat.1006230.s007.TIF]

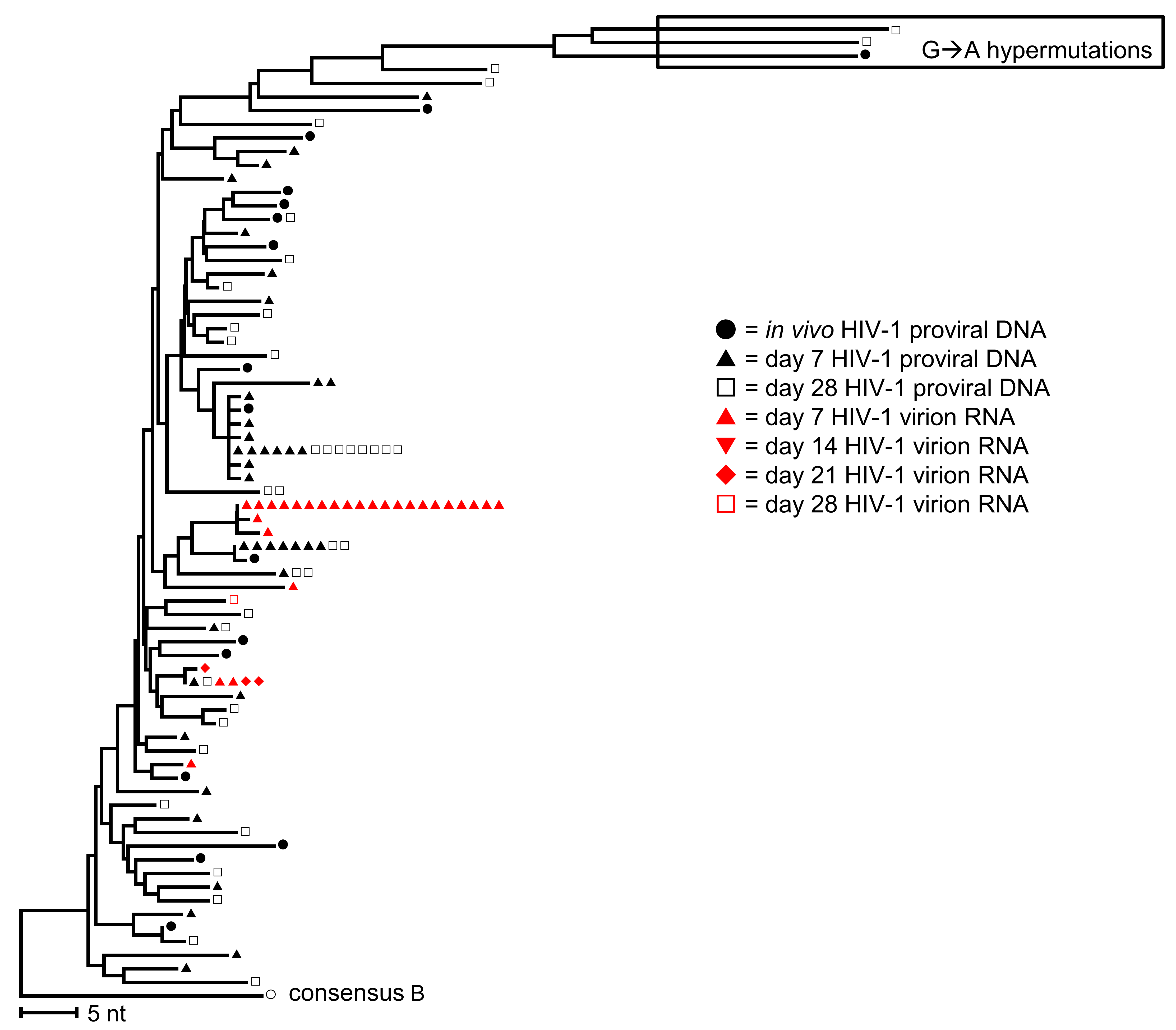

Supplement: S8 Fig — Sequences were rooted to a consensus sequence of HIV subtype B. The tree was constructed using the neighbor-joining p-distance method. Hypermutant sequences are in boxes. (TIF) [file ppat.1006230.s008.TIF]

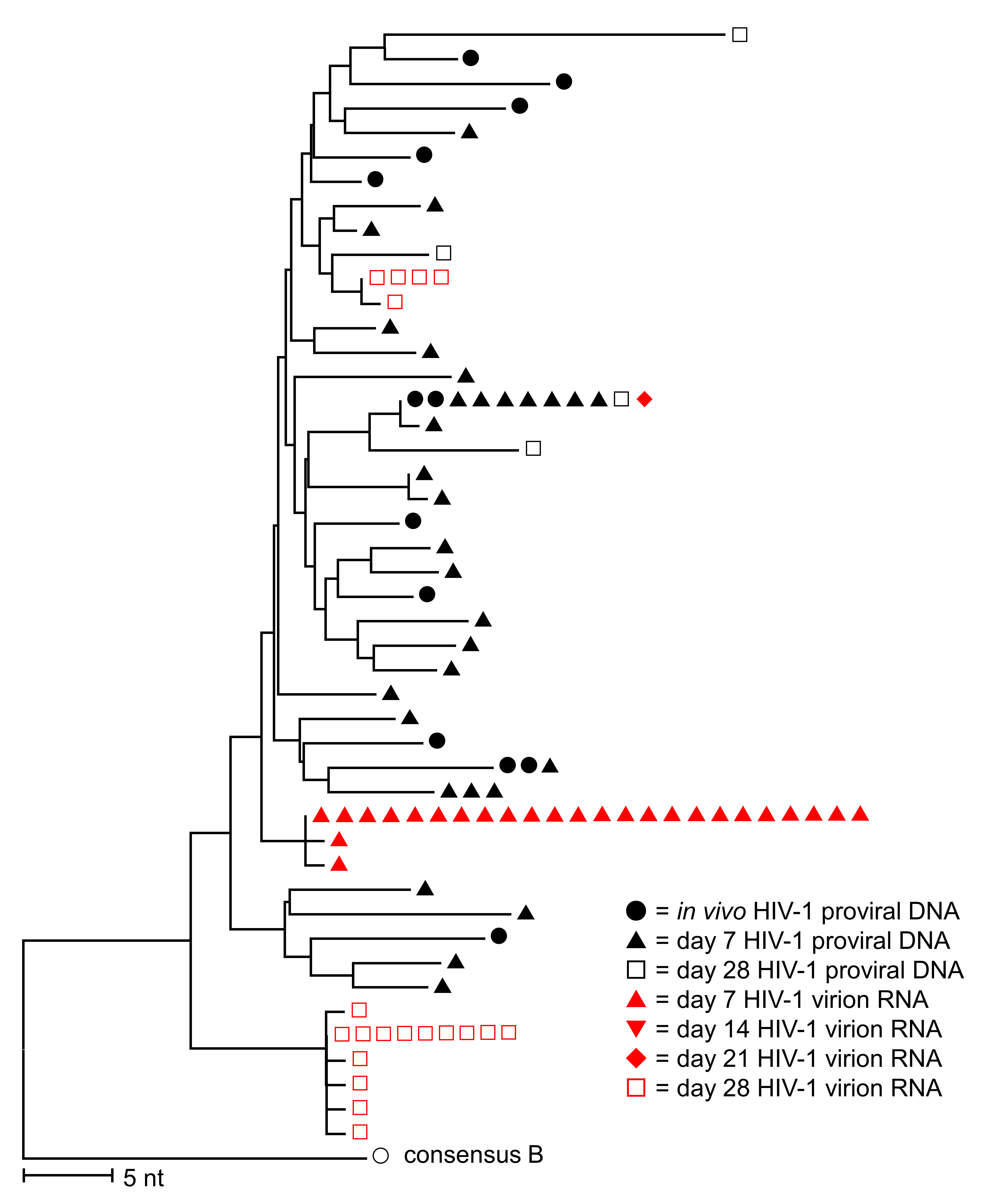

Supplement: S9 Fig — Sequences were rooted to a consensus sequence of HIV subtype B. The tree was constructed using the neighbor-joining p-distance method. Hypermutant sequences are in boxes. (TIF) [file ppat.1006230.s009.TIF]
