# Supplementary material for: Ex vivo activation of CD4+ T-cells from donors on suppressive ART can lead to sustained production of infectious HIV-1 from a subset of infected cells
Source: PLoS Pathog. 2017 Feb 22;13(2):e1006230. doi: 10.1371/journal.ppat.1006230 (PMC5338860; doi:10.1371/journal.ppat.1006230)
Supplement: S1 Table — Different proviral population outcomes are quantified for experiments with PBMC. Each outcome is calculated as either 1) the frequency of proviruses displaying a given outcome relative to the total number of unique proviral sequences observed over the entire duration of cell culture, or 2) the frequency of unique virion sequences displaying a given outcome relative to the total number of unique virion sequences observed over the entire duration of cell culture. (DOCX) [file ppat.1006230.s010.docx]

**S1 Table. Proviral expression and dynamics in PBMC after sequential stimulation.**

|  |  | **Donor 1** | **Donor 5** |
| --- | --- | --- | --- |
| **% of Unique Virion Sequences** | **Virions detected only after first stimulation** | **57.9%** | **50.0%** |
|  | **Virions detected only after second stimulation** | **21.1%** | **50.0%** |
|  | **Virions detected with both stimulations** | **21.1%** | **Not detected** |
|  | ***Ex vivo* persistence or expansion of intact, inducible proviruses** | **10.5%** | **Not**  **done** |
| **% of Unique Proviral Sequences** | ***Ex vivo* expansion of non-induced proviruses** | **1.2%** | **< 3.0%** |
|  | ***Ex vivo* expansion of inducible proviruses** | **1.2%** | **< 3.0%** |
