# Supplementary material for: Ex vivo activation of CD4+ T-cells from donors on suppressive ART can lead to sustained production of infectious HIV-1 from a subset of infected cells
Source: PLoS Pathog. 2017 Feb 22;13(2):e1006230. doi: 10.1371/journal.ppat.1006230 (PMC5338860; doi:10.1371/journal.ppat.1006230)
Supplement: S2 Table — The probability of detecting two identical p6-PR-RT sequences assuming no clonal expansion had occurred was calculated for each experiment using the binomial distribution based on the average pairwise distance (APD) of all obtained proviral sequences for each experiment. Hypermutant sequences were excluded from analysis. (DOCX) [file ppat.1006230.s011.docx]

**S2 Table. Probability estimate of detecting two identical proviral sequences.**

| Donor | Cell Type | Number of Proviral Sequences | Length of trimmed amplicon (bp) | APD of All Proviral Sequences (%) | Probability of detecting two identical proviral sequences (%) |
| --- | --- | --- | --- | --- | --- |
| 1 | Total CD4^+^ T-cells | 102 | 1516 | 2.1 | < 1x10^-8^ |
| 1 | Total CD4^+^ T-cells (repeat) | 81 | 1516 | 2.2 | < 1x10^-8^ |
| 1 | PBMC | 78 | 1516 | 2.1 | < 1x10^-8^ |
| 2 | Total CD4^+^ T-cells | 105 | 1516 | 1.4 | < 1x10^-3^ |
| 3 | Total CD4^+^ T-cells | 87 | 1510 | 1.5 | < 1x10^-4^ |
| 4 | Total CD4^+^ T-cells | 104 | 1507 | 1.7 | < 1x10^-6^ |
| 5 | Total CD4^+^ T-cells | 89 | 1517 | 1.5 | < 1x10^-5^ |
| 5 | PBMC | 47 | 1516 | 1.2 | < 1x10^-2^ |
