# Supplementary material for: Ex vivo activation of CD4+ T-cells from donors on suppressive ART can lead to sustained production of infectious HIV-1 from a subset of infected cells
Source: PLoS Pathog. 2017 Feb 22;13(2):e1006230. doi: 10.1371/journal.ppat.1006230 (PMC5338860; doi:10.1371/journal.ppat.1006230)
Supplement: S3 Table — Hypermutant sequences were excluded from analysis. N/A = not applicable because < 5 sequences recovered. (DOCX) [file ppat.1006230.s012.docx]

**S3 Table. Average Pairwise Distances (APD) of Proviral DNA Sequences.**

| Donor | Cell Type | APD % of Day 0 proviral sequences (number of sequences) | APD % of Day 7 proviral sequences (number of sequences) | APD % of Day 28 proviral sequences (number of sequences) |
| --- | --- | --- | --- | --- |
| 1 | CD4+ T-cells | 2.1 (37) | 2.1 (29) | 2.1 (36) |
| 1 | CD4+ T-cells (repeat) | 2.1 (27) | 2.2 (29) | 2.2 (25) |
| 1 | PBMC | 2.1 (14) | 2.1 (38) | 2.1 (26) |
| 2 | CD4+ T-cells | 1.5 (30) | 1.4 (37) | 1.4 (38) |
| 3 | CD4+ T-cells | 1.5 (32) | 1.6 (29) | 1.5 (26) |
| 4 | CD4+ T-cells | 1.5 (37) | 1.6 (32) | 1.8 (35) |
| 5 | CD4+ T-cells | 1.9 (15) | 1.1 (36) | 1.6 (38) |
| 5 | PBMC | 1.2 (13) | 1.0 (30) | N/A (4) |
