# Supplementary material for: Ex vivo activation of CD4+ T-cells from donors on suppressive ART can lead to sustained production of infectious HIV-1 from a subset of infected cells
Source: PLoS Pathog. 2017 Feb 22;13(2):e1006230. doi: 10.1371/journal.ppat.1006230 (PMC5338860; doi:10.1371/journal.ppat.1006230)
Supplement: S4 Table — Hypermutant sequences were excluded from analysis. N/A = not applicable because < 5 sequences recovered. (DOCX) [file ppat.1006230.s013.docx]

**S4 Table. Average Pairwise Distances (APD) of supernatant viral RNA sequences.**

| Donor | Cell Type | APD % of Day 7 virion sequences (number of sequences) | APD % of Day 14 virion sequences (number of sequences) | APD % of Day 21 virion sequences (number of sequences) | APD % of Day 28 virion sequences (number of sequences) |
| --- | --- | --- | --- | --- | --- |
| 1 | Total CD4^+^ T-cells | 1.9 (24) | 1.3 (29) | 1.0 (25) | 1.4 (24) |
| 1 | Total CD4^+^ T-cells (repeat) | 2.1 (28) | 1.3 (8) | 1.1 (42) | 1.0 (9) |
| 1 | PBMC | 1.8 (25) | 1.6 (14) | N/A (2) | 1.8 (24) |
| 2 | Total CD4^+^ T-cells | 1.3 (28) | 1.1 (22) | 1.2 (8) | 1.0 (27) |
| 3 | Total CD4^+^ T-cells | 1.3 (22) | 0.9 (22) | 1.1 (28) | 1.2 (23) |
| 4 | Total CD4^+^ T-cells | 1.3 (22) | 1.8 (24) | 1.3 (23) | 1.4 (22) |
| 5 | Total CD4^+^ T-cells | 0.3 (27) | N/A (0) | N/A (3) | N/A (1) |
| 5 | PBMC | 0.0 (25) | N/A (0) | N/A (1) | 0.5 (17) |
